# High Versatility of IPP and DMAPP Methyltransferases Enables Synthesis of C_6_, C_7_ and C_8_ Terpenoid Building Blocks

**DOI:** 10.1002/cbic.202200091

**Published:** 2022-06-07

**Authors:** Laura Drummond, Parab J. Haque, Binbin Gu, Julia S. Jung, Hendrik Schewe, Jeroen S. Dickschat, Markus Buchhaupt

**Affiliations:** ^1^ Microbial Biotechnology DECHEMA Research Institute Theodor-Heuss-Allee 25 60486 Frankfurt am Main Germany; ^2^ Department of Microbiology and Biochemistry Hochschule Geisenheim University Von-Lade-Strasse 1 65366 Geisenheim Germany; ^3^ University of Bonn Kekulé-Institute for Organic Chemistry and Biochemistry Gerhard-Domagk-Straße 1 53121 Bonn Germany

**Keywords:** DMAPP, IPP, non-canonical terpenes, terpenoids, transferases

## Abstract

The natural substance class of terpenoids covers an extremely wide range of different structures, although their building block repertoire is limited to the C_5_ compounds DMAPP and IPP. This study aims at the characterization of methyltransferases (MTases) that modify these terpene precursors and the demonstration of their suitability for biotechnological purposes. All seven enzymes tested accepted IPP as substrate and altogether five C_6_ compounds and six C_7_ compounds were formed within the reactions. A high selectivity for the deprotonation site as well as high stereoselectivity could be observed for most of the biocatalysts. Only the enzyme from *Micromonospora humi* also accepted DMAPP as substrate, converting it into (2*R*)‐2‐methyl‐IPP *in vitro. In vivo* studies demonstrated the production of a C_8_ compound and a hydride shift step within the MTase‐catalyzed reaction. Our study presents IPP/DMAPP MTases with very different catalytic properties, which provide biosynthetic access to many novel terpene‐derived structures.

## Graphical abstract

**Nearly all known terpenes** are built from the two C5 precursors DMAPP and IPP. This work characterizes several bacterial DMAPP and IPP methyltransferases that convert these building blocks in C_6_, C_7_ and C_8_ prenyl pyrophosphates with selectivity on deprotonation sites and stereoselectivity.

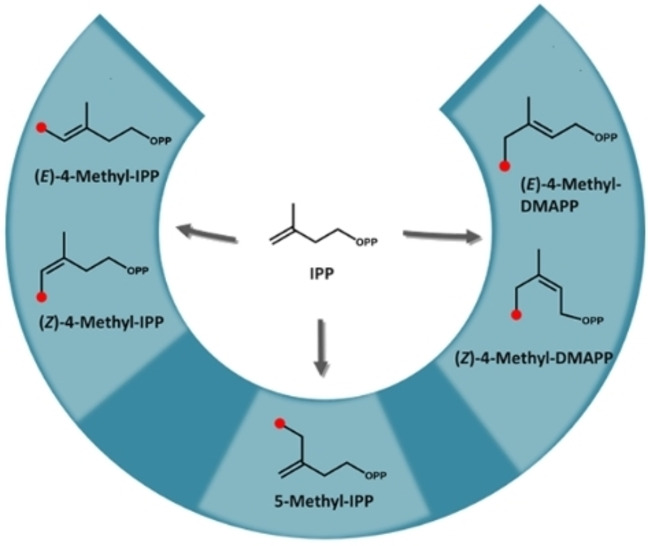

## Introduction

Terpenes, also known as isoprenoids, are one of the best‐studied classes of natural substances with more than 70,000 known structures.^[1]^ The substance group has been investigated in enormous detail with regard to structural diversity, biosynthesis and the functions of individual molecules. This is due to their occurrence in plant oils in relatively high concentrations and their use by humans as, for example, flavoring agents, dyes, pharmaceuticals and even structural materials like natural rubber and gutta‐percha. Despite the enormous structural diversity, there are almost exclusively only two basic building blocks, IPP (isopentenyl pyrophosphate) and DMAPP (dimethylallyl pyrophosphate), from which all terpenoid backbones are built up. The two C_5_ intermediates can be condensed by prenyltransferases to form longer pyrophosphate chains, which can be converted by terpene synthases into very diverse acyclic, cyclic or polycyclic structures. The number of carbon atoms in terpene synthase substrates and products is usually a multiple of 5. Otto Wallach recognized this law more than 120 years ago^[2]^ and in 1953 Leopold Ružička described it in the so‐called (biogenetic) isoprene rule.^[3]^ The latter established the dogma of the cellular repertoire of pyrophosphate‐containing terpene intermediates with only multiples of 5 (10, 15, 20, etc.) carbon atoms. However, it also implies the possibility of further changes in the terpene carbon frameworks through for example terpene synthase‐catalyzed cleavage, as in the case of geosmin,^[4]^ and through subsequent acetylation or other enzymatic modifications. Hitherto two exceptions to the isoprene rule are known, showing that cells have developed mechanisms for the synthesis of further isoprenoid pyrophosphates generating an increase in the structural diversity of this natural compound class. The first exception was demonstrated with the discovery of different derivatives of the insect juvenile hormone containing extra methyl groups,[^5^, ^6^] which in turn led to a closer inspection of the IPP‐ and DMAPP‐delivering mevalonate pathway in such organisms. It was shown that the respective pathway surprisingly accepts propionyl‐CoA in addition to the normal starter unit acetyl‐CoA, and in this way forms IPP and DMAPP derivatives with an additional methyl group.[^7^, ^8^, ^9^, ^10^] In addition to the juvenile hormone derivatives, other terpene structures with additional methyl groups were then found in insects, which were usually referred to as homoterpenes in the literature (e. g. Ref. [11–14]).

The second exception involves a fundamentally different concept for introducing additional carbon atoms into terpene precursors, which is present in certain bacteria and was discovered during the elucidation of the biosynthetic pathway of the C_11_ terpenoid 2‐methylisoborneol.[^15^, ^16^, ^17^] In this example the C_10_ terpene precursor GPP (geranyl pyrophosphate) is methylated by an MTase (methyltransferase) in a SAM (S‐adenosyl‐methionine)‐dependent reaction at C2. The resulting 2‐methyl‐GPP is then converted into 2‐methylisoborneol or 2‐methylenebornane. In *Serratia plymuthica* the C_15_ terpene precursor FPP (farnesyl pyrophosphate) can be converted into a cyclic C_16_ pyrophosphate (presodorifen pyrophosphate) by another MTase. A terpene synthase afterwards catalyzes the transformation of the unusual intermediate into the bicyclic terpene sodorifen.^[18]^

**Figure 1:**
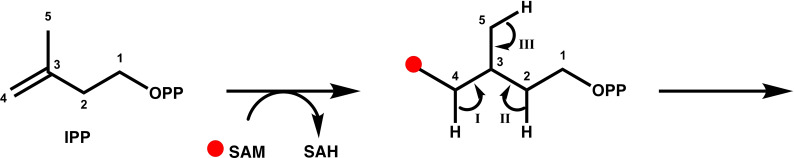
Schematic reaction mechanism catalyzed by IPP MTases. The addition of a methyl group at C4 (indicated by a red dot) generates a carbocation that can be stabilized by different deprotonation pathways, indicated in the picture with roman numerals.

Moreover, SAM‐dependent IPP MTases have been discovered in three different biosynthetic contexts. They can convert the basic terpene building block IPP into different C_6_ and C_7_ prenyl pyrophosphates and thereby provide exactly those intermediates that also occur in insects as unusual mevalonate pathway products. In *Rhodococcus fascians* two prenyl pyrophosphate MTases provide different methylated prenyl pyrophosphates for the synthesis of unusual cytokinins.^[19]^ The functions of the enzyme called MT2 were determined. It transfers a methyl group to the C4 atom of IPP, thereby synthesizing 4‐methyl‐DMAPP. Furthermore it synthesizes the C_7_ compound 4,5‐dimethyl‐DMAPP together with MT1. Another IPP methyltransferase was discovered in *Streptomyces argenteolus* as a (*Z*)‐4‐methyl‐IPP‐synthesizing enzyme, which is part of the longestin biosynthetic pathway.^[20]^ The C_6_ compound is introduced with high positional accuracy at two positions of a GGPP (geranylgeranyl pyrophosphate) derivative, from which the meroterpenoid longestin is finally produced. The third functionally characterized IPP MTase is encoded in an operon in *Streptomyces monomycini*,^[21]^ which furthermore encompasses genes encoding a putative prenyl transferase and a putative terpene synthase. The MTase was characterized in detail and shown to synthesize not only (*E*)‐4‐methyl‐IPP as main product, but also a number of other C_6_ and C_7_ prenyl pyrophosphates, even though the structure of the terpene product of the three enzyme pathway remains unknown. MTases, that modify the second major building block of terpenes, DMAPP, have not been discovered in nature up to now. The identification of several different C_6_ products in the *S. monomycini* IPP MTase reaction points to a carbocation intermediate, which is in line with the mechanism that has been proposed for the GPP MTase, electrophilic attack of SAM on the double bond to form a carbocation and deprotonation.^[22]^ Depending on the position of a general base, proton abstraction can occur at one of the carbon atoms neighboring the carbocation (Figure 1). In case of deprotonations at C4 or at C2, two different stereoisomers with *E*‐ or *Z*‐configured double bond may be formed in each case.

Due to their ability to extend the chemical space of terpenes, the GPP MTase has been integrated into cellular biosynthesis pathways with different strategies aiming at production of novel terpene products.[^23^, ^24^] For more detailed information about strategies of terpene structure diversification via MTases or other concepts, the reader is referred to recent reviews in this field.[^25^, ^26^, ^27^]

This study aimed at a comparison of the three known IPP MTases and the isolation and characterization of novel C_5_ prenyl pyrophosphate MTases with different properties. Detailed descriptions of substrates and products for this group of enzymes and demonstration of their *in vivo* performance will enable diverse biotechnological applications.

## Results and Discussion

### Theoretical structure diversification potential for terpene precursors via IPP MTases

**Figure 2:**
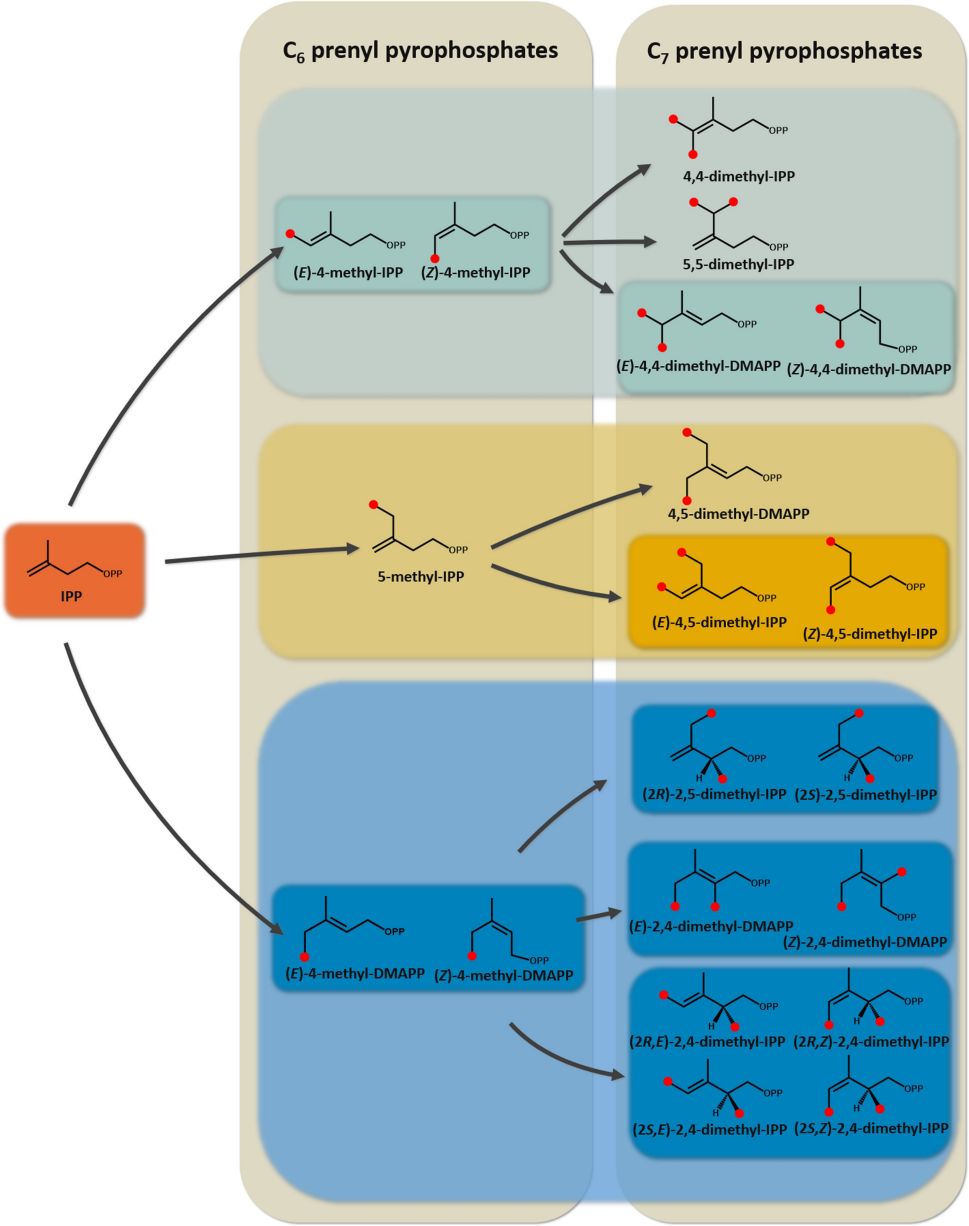
Overview of all C_6_ and C_7_ prenyl pyrophosphates accessible by one methylation or two subsequent methylations starting from IPP via the mechanisms shown in Figure 1. Red dots indicate the positions of the newly introduced methyl groups. Boxes are used to indicate groups of stereoisomers.

Three IPP MTases have been characterized so far with respect to the products they form.[^19^, ^20^, ^21^] Although several different products were already shown to be formed by these enzymes from IPP in *in vitro* reactions, the number of potentially accessible methylated and dimethylated IPP derivatives is much higher. Figure 2 depicts the different C_6_ and C_7_ prenyl pyrophosphates that should be accessible via the mechanisms shown in Figure 1. After methylation at C4, formation of five different products is possible, including two pairs of diastereomers. A second methylation step using the C_6_ compounds as substrates could yield up to 15 different C_7_ products. However, one must consider that the C_7_ products in the blue box can only be formed if the enzyme transfers the second methyl group to C2 of the corresponding C_6_ precursors.

### Selection of different potential C_5_ prenyl pyrophosphate MTase sequences

In order to identify novel C_5_ prenyl pyrophosphate MTases, we searched for protein sequences with similarity to the IPP MTases from *S. monomycini* ^[21]^ and *S. argenteolus* ^[20]^ and analyzed the genes neighboring the MTase‐encoding genes. To increase the potential for identification of enzymes with novel properties, we selected candidates whose operons clearly showed differences with regard to the encoded proteins. We selected four operons, which differed clearly in terms of the number of encoded proteins, but also by the enzymatic functions, which were annotated for the proteins. By this way four enzymes from different species were chosen. The protein WP_091072690 from *Micromonospora humi* (humMT) is encoded in a putative operon together with a putative aminotransferase, a dehydrogenase and a prenyltransferase enzyme. WP_030281021 from *Streptomyces catenulae* (catMT) is presumably encoded in an operon containing also an oxidoreductase‐ and a prenyltransferase‐encoding gene. In *Amycolatopsis azurea* the WP_005164808 (azuMT)‐encoding gene is possibly part of an operon that contains three more genes, all of which putatively encode prenyltransferases. A completely different operon was chosen from *Frankia* sp., in which the putative MTase sequence WP_076843458 (fraMT) is encoded adjacent to three other probable MTase‐encoding genes and other proteins with no obvious relation to terpene biosynthesis pathways. All four putative operons do not encode terpene synthase genes. The operon structures of the respective genes have been discussed already in the respective publications and are shown again in Figure S2.

To be able to directly compare the functions of the newly discovered putative prenyl pyrophosphate MTases with those of the already characterized IPP MTases from *R. fascians*,^[19]^ *S. argenteolus* ^[20]^ and *S. monomycini*,^[21]^ we decided to include these three enzymes in our experiments.

### Product determination for the selected MTases using IPP as substrate

**Figure 3:**
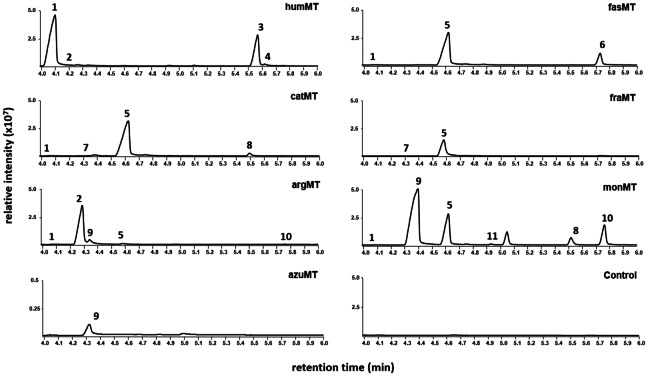
Product spectrum determination for different SAM‐dependent IPP MTases. GC chromatograms (TIC) demonstrating the products of humMT, fasMT, catMT, fraMT, argMT, monMT and azuMT with IPP as prenyl pyrophosphate substrate. After heterologous expression in *E. coli* and purification, the proteins were incubated with IPP and SAM as described in the material and methods section. The control reaction contained no enzyme. After a phosphatase reaction step, the alcohol derivatives of respective prenyl pyrophosphates were detected by headspace‐SPME‐GC‐MS analyses and identified by comparison of the obtained mass spectra with mass spectra of reference compounds (Figure S4A–S4K) as well as retention times (Figure S3). Shown are representative chromatograms after several repetitions of the experiments.

In order to characterize the substrates and products of the selected enzymes, we first tested their ability to convert IPP, as three of them were already shown to accept IPP as substrate.[^19^, ^20^, ^21^] Purified enzymes were incubated with SAM and IPP, and the products in form of terpene alcohols, after phosphatase treatment, were detected via headspace‐GC‐MS using SPME fibers (Figure 3). Altogether eleven different terpene alcohols could be detected, from which ten could be assigned to specific structures shown in Figure S3. In the reaction with humMT, 5‐methylisoprenol (**1**, Figure S3) was the main C_6_ product accompanied by traces of (*Z*)‐4‐methyl‐isoprenol (**2**, Figure S3). The main C_7_ product could be identified as one of the stereoisomers of 4,5‐dimethyl‐isoprenol (**3**, Figure S3), whereas only small amounts of the other stereoisomer of 4,5‐dimethyl‐isoprenol (**4**, Figure S3) could be detected. Therefore, although methylation takes place at carbon atom 4, deprotonation at carbon 5 leads to the main product 5‐methyl‐IPP, whereas the IPP MTases from *S. monomycini*, *S. argenteolus* and *R. fascians* favor deprotonation at carbon atom 4[^20^, ^21^] or 2,^[19]^ respectively. The deprotonation site selectivity of humMT seems to be very high, as only traces of **2** could be detected. Moreover, the stereospecificity of the deprotonation at C4 in the second methylation step is also high, as demonstrated by low amounts of **4** in relation to the intensity of **3**. fasMT forms prenyl pyrophosphates that correspond to the C_6_ compound (*E*)‐4‐methyl‐prenol (**5**, Figure S3) and the unknown C_7_ compound **6**. Although Radhika *et al*. did not investigate stereochemistry aspects regarding *E* or *Z* configuration of the product, they also showed 4‐methyl‐DMAPP to be the product of IPP methylation by this enzyme.^[19]^ The C_7_ compound **6** was not reported in the mentioned publication and we could not detect it in any other enzyme reaction either. Furthermore, its structure remains unclear, as we could not find a perfect match of its retention time and its mass spectrum (Figure S4F) to that of 4,5‐dimethyl‐prenol or to those of any of the stereoisomers present in the compound mixtures of 4,4‐dimethyl‐prenol, 4,5‐dimethyl‐isoprenol, 2,5‐dimethyl‐isoprenol, 2,4‐dimethyl‐prenol or 2,4‐dimethyl‐isoprenol.

The high selectivity for deprotonation at C2 is also a characteristic of catMT, as it yields almost exclusively 4‐methyl‐DMAPP detected by the release of **5**, whose corresponding pyrophosphate seems to be not accepted as substrate for a second methylation reaction by the enzyme. Several enzymes including catMT produce only the *E*‐form of 4‐methyl‐DMAPP, as demonstrated by the appearance of **5**, but no or negligible amounts of (*Z*)‐4‐methyl‐prenol (**7**, Figure S3). The high *E*/*Z* stereoselectivity in the deprotonation step points to a strong conformational fixation of the substrate IPP in the enzyme's active site. The appearance of 4,4‐dimethyl‐prenol (**8**, Figure S3) in small amounts in the reaction with catMT can only be explained by methylation of 4‐methyl‐IPP (corresponding alcohols **2** or **9**), which is surprising, because the corresponding alcohol 3‐methylpent‐3‐en‐1‐ol was not detected. This may point to an efficient and rapid further conversion of 4‐methyl‐IPP by a second methylation step. The chromatogram of the reaction with fraMT from *Frankia* sp. also shows nearly exclusively **5**, but lacks **8** possibly because this enzyme does not generate any 4‐methyl‐IPP.

ArgMT was found to be highly selective in the formation of (*Z*)‐4‐methyl‐IPP as already described^[20]^ and shown by the appearance of **2**. However, the detection of minor amounts of (*E*)‐4‐methyl‐isoprenol (**9**, Figure S3) shows that deprotonation does not occur in a completely stereoselective way. The already characterized IPP methyltransferase monMT^[21]^ shows high stereoselectivity for the product (*E*)‐4‐methyl‐IPP, as only **9**, but not **2** could be detected here. In a former study, small amounts of **2** were formed by the enzyme.^[21]^ Furthermore, this protein is unique in the low selectivity for the deprotonation site, which is shown by substantial formation of **9** (deprotonation at C4) and **5** (deprotonation at C2). This promiscuity can also be seen for the second methylation reaction, as the appearance of compounds **8**, 4,4‐dimethyl‐isoprenol (**10**, Figure S3) and 5,5‐dimethyl‐isoprenol (**11**, Figure S3) results from deprotonations at C2, C4 or C5 (Figure 3 and Ref. [21]). In reactions with azuMT, only very low amount of **9** was found.

Altogether, we found a broad diversity for the reactions catalyzed by the tested IPP MTases. Whereas monMT is relatively promiscuous with regard to the deprotonation site, most of the enzymes showed high selectivity for this aspect. The ability to introduce a second methyl group also seems to differ between the different catalysts. The high stereoselectivity of deprotonation at C2 or C4 make the enzymes attractive for production of C_6_ and C_7_ alcohols with high stereoisomer purity.

### DMAPP is mono‐methylated at C2 by humMT

**Figure 4:**
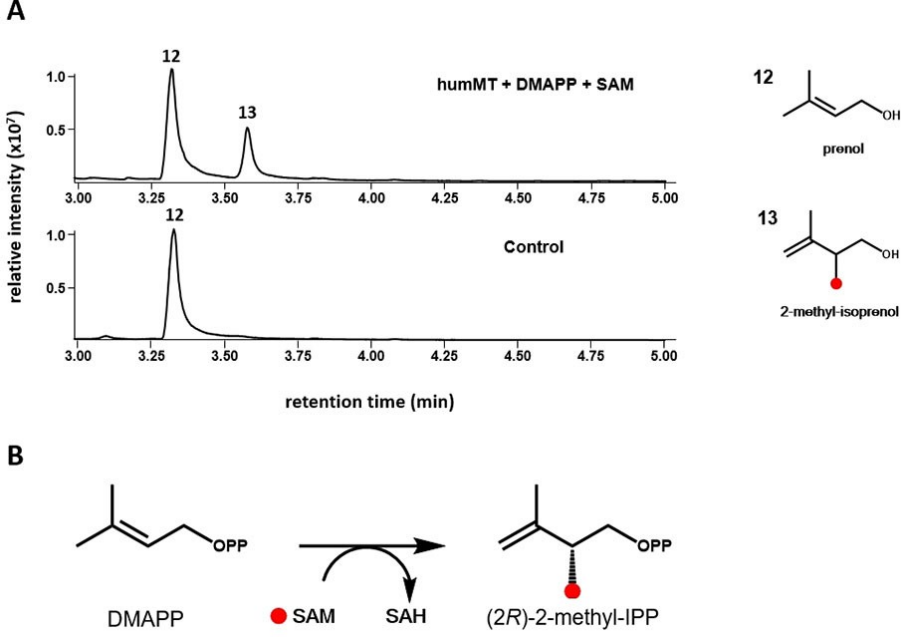
Identification of the DMAPP C2 MTase activity of humMT. A) The upper chromatogram represents the HS‐SPME‐GC/MS chromatogram (TIC) of the humMT‐catalyzed SAM‐dependent conversion of DMAPP. The lower chromatogram shows the corresponding analysis of the reaction without enzyme. Identification of **13** as 2‐methyl‐isoprenol and **12** as prenol was performed by comparison of retention indexes and mass spectra with the respective reference compounds (Figure S4L and S4M). The deduced DMAPP conversion by the enzyme is depicted in the figure. The absolute configuration of **13** was elucidated by GC on a chiral cyclodextrin stationary phase connected to an MS detector, and by comparison of the retention time of the enzymatically formed product with the ones from both enantiomers (Figure S2). The red dot indicates the position of the newly introduced methyl group. B) Reaction scheme for enantioselective C2‐methylation of DMAPP by humMT. The red dot indicates the position of the newly introduced methyl group.

**Figure 5:**
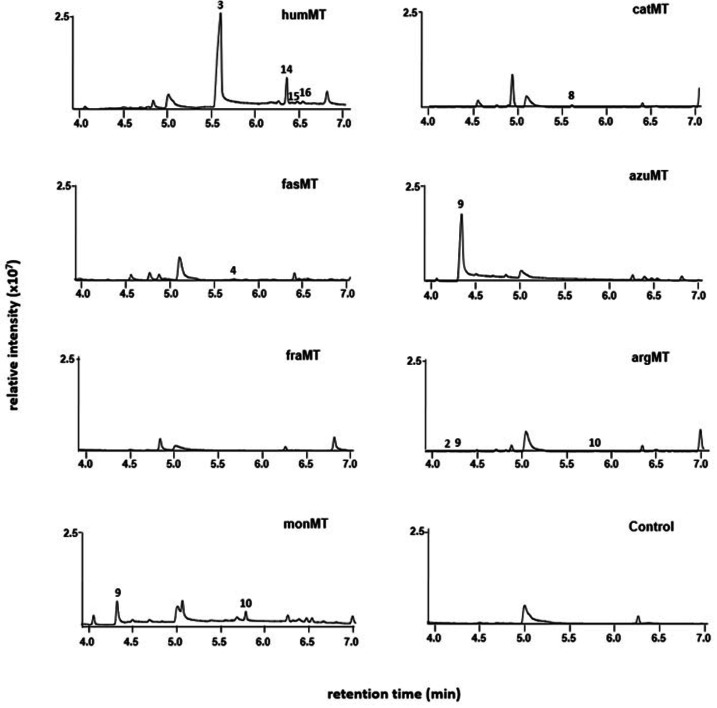
*In vivo* synthesis of methylated IPP and DMAPP derivatives in *E. coli* strains expressing one of the prenyl pyrophosphate MTase genes. *E. coli* MG1655 (DE3) Δ*recA* Δ*endA* Δ*tnaA* strains containing the mevalonate pathway plasmid pLD‐03 and additionally containing pPJH‐1 (humMT*)*, pPJH‐2 (fasMT), pPJH‐3 (catMT), pPJH‐4 (fraMT), pPJH‐5 (azuMT), pPJH‐6 (argMT) or pMK‐24 (monMT) were cultivated as described in the material and methods section. The strain used for the control experiment contained pLD‐03 and pET28a(+). The alcohol derivatives of respective prenyl pyrophosphates were detected by headspace‐SPME‐GC‐MS analyses and products were identified by comparison of the obtained mass spectra with mass spectra of reference compounds (Figure S4) as well as retention times (Figure S3). Shown are representative TIC chromatograms after several repetitions of the experiments.

As we wanted to test the substrate promiscuity of all MTases, reactions with DMAPP and GPP were performed and analyzed for the appearance of methylated conversion products. None of the enzymes was able to methylate GPP, as we could not detect 2‐methylgeraniol or any other product in respective reactions (data not shown). Nonetheless, DMAPP was accepted as substrate by the MTase from *M. humi* (humMT), but not by any of the other enzymes. Figure 4A shows that besides prenol (**12**), the dephosphorylation product of DMAPP, a C_6_ compound could be detected in the respective reaction sample, which was identified as 2‐methyl‐isoprenol (**13**), the dephosphorylation product of 2‐methyl‐IPP. To elucidate the absolute configuration of **13**, GC‐MS analyses of the product in comparison to both synthetic enantiomers of **13** were performed using a chiral stationary phase (for a description of the synthesis cf. SI). The data in Figure S2 shows that the enzyme is highly enantioselective and produces only (2*R*)‐**13** (Figure 5B).

The catalyzed reaction is analogous to the methylation of GPP at C2 by GPP MTase with regard to the position, but the position of the double bond in the products is different. Whereas the carbocation in the GPP MTase reaction is quenched by deprotonation at C2, deprotonation of the reaction intermediate in the *M. humi*‐MTase‐catalyzed DMAPP methylation reaction takes place at C4. To our knowledge, GPP MTases have not been tested for acceptance of DMAPP so far. A previously performed rational protein engineering approach aimed at adaptation of the GPP MTase towards methylation of other prenyl pyrophosphates.^[28]^ The study demonstrated SAM consumption by the G202A variant of the *S. coelicolor* GPP MTase in the presence of DMAPP, but failed to identify methylated products. Therefore, humMT is the first enzyme shown so far to have DMAPP methylation activity. The fact that the enzyme from *M. humi* is able to methylate IPP and DMAPP suggests that the transferable methyl group of bound SAM must be in close proximity not only to the 3,4 π bond of IPP, but also to the 2,3 π bond of DMAPP.

### In vivo production of C_6_, C_7_ and C_8_ prenyl pyrophosphates by the different MTases

After successfully demonstrating the enzymatic formation of different C_6_ and C_7_ prenyl pyrophosphates with the selected MTases *in vitro*, we aimed at testing their performance in a cellular environment. Therefore, we expressed the MTases in an *E. coli* strain, which was engineered towards high IPP and DMAPP production. The headspace of the cultures was analyzed via SPME‐GC‐MS, which led to the identification of different C_6_, C_7_ and C_8_ alcohols (Figure 5). The strain expressing humMT released the C_7_ compound **3**, which had been found as one of the main products in *in vitro* experiments, but did not produce the C_6_ compound **1**. Furthermore, the strain released terpene alcohols, whose mass spectra showed them to be C_8_ compounds. Figure S4N shows the mass spectrum of the main C8 terpene alcohol (**14**). Expression of catMT resulted in the release of very low amounts of **8**, whereas the main product from the enzyme reaction analysis (**5**) was not found here. In the culture headspace of the strain expressing the MTase fasMT, hardly detectable amounts of **4** could be identified, whereas the main products from the *in vitro* reaction (**5** and **7**) were not found. In case of azuMT, the respective strain formed only **9**, the only compound identified also in *in vitro* experiments. No methylated terpene alcohols could be detected in the culture headspace of strains expressing fraMT. With the culture of the strain expressing argMT, only minor amounts of **2**, **9** and **10** could be detected. The strain expressing monMT released the C_6_ compound **9** and the C_7_ compound **10**, which were also main products in the *in vitro* experiments.

The fact that trimethylated prenyl pyrophosphates were synthesized by the MTase from *M. humi* only in the cellular system might have been caused by higher stability or activity of the enzyme in the cells or higher SAM availability. However, as the chromatograms only represent the compounds present in the culture headspace, the data in Figure 5 must be interpreted very carefully in general. The possibility exists, that the different prenyl pyrophosphates might be hydrolyzed by endogenous *E. coli* phosphatases with very different rates. Therefore, some prenyl pyrophosphates might accumulate in the cells, although the corresponding alcohols are not detectable in the chromatograms. Another possibility for the nonappearance of MTase products in our analysis is the incorporation of the prenyl pyrophosphates in e. g. hexadecaprenol derivatives to different extents. Another caveat of the *in vivo* data is that these enzymes are not in their native host and product determinations, yields, and regulation may all change based on their environments.

If an MTase enzyme synthesizes not only one unusual prenyl pyrophosphate intermediate, it will be difficult to integrate MTases in e. g. a sesquiterpene production strain to yield one specific C_16_ or C_17_ product. In general it will probably be necessary to engineer the MTases for high product selectivity and/or to engineer downstream enzymes in the biosynthetic pathway for high selectivity. AzuMT seems to be an attractive exception, as the *in vitro* and *in vivo* experiments demonstrated the production of almost only **9**.

### Formation of the main C_8_ prenyl pyrophosphate by humMT includes a hydride shift

**Figure 6:**
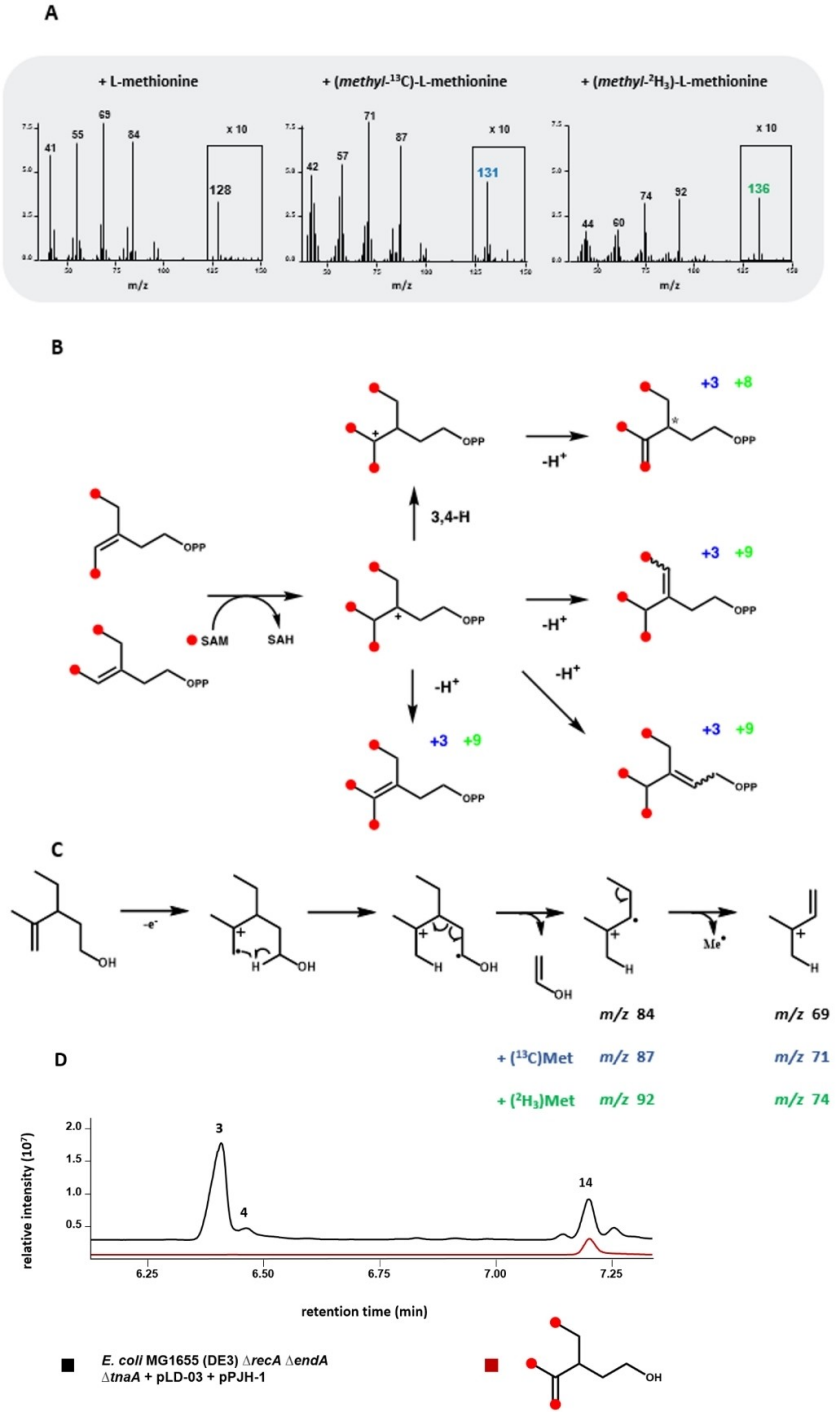
Identification of the main C_8_ compound (14) produced by humMT *in vivo*. A) EI mass spectra of the main C8 terpene alcohol produced by *E. coli* MG1655 (DE3) Δ*recA* Δ*endA* Δ*tnaA*+pLD‐03+pPJH‐1 cultivated in medium supplemented with L‐methionine (left), (*methyl*‐^13^C)‐L‐methionine (middle) or (*methyl*‐^2^H_3_)‐L‐methionine. The fragment signal intensity in the box was magnified by a factor of 10 to show the mass of the molecular ion B) Proposed reaction paths for methylation of 4,5‐dimethyl‐IPP by humMT. Red dots indicate the positions of the newly introduced methyl groups. Blue numbers indicate the expected mass shift for each potential product after supplementation with (*methyl*‐^13^C)‐L‐methionine. Green numbers indicate the expected mass shift for each potential product after supplementation with (*methyl*‐^2^H_3_)‐L‐methionine. The asterisk at C3 indicates a chiral center. C) Proposed EI‐MS fragmentation of 3‐ethyl‐4‐methylpent‐4‐en‐1‐ol, the suggested structure of **14**. The expected fragment ions for the labelling experiments with (*methyl*‐^13^C)‐L‐methionine and (*methyl*‐^2^H_3_)‐L‐methionine are shown in red and blue, respectively. D) Comparative GC analysis for the products of *E. coli* MG1655 (DE3) Δ*recA* Δ*endA* Δ*tnaA*+pLD‐03+pPJH‐1 culture and for the reference compound 3‐ethyl‐4‐methylpent‐4‐en‐1‐ol. Red dots indicate the positions of the newly introduced methyl groups. Respective mass spectra comparisons are shown in Figure S4N.

Aiming at structure elucidation of the main C_8_ prenyl pyrophosphate (pyrophosphate of **14**) produced by the MTase from *M. humi*, we performed feeding experiments with (*methyl*‐^13^C)‐L‐methionine or (*methyl*‐^2^H3)‐L‐methionine to get additional information about the fate of the three methyl groups. As expected, we observed a mass shift of +3 for the molecular ion of **14** after supplementation of the culture medium of the humMT‐expressing *E. coli* strain with (*methyl*‐^13^C)‐L‐methionine (Figure 6A), which is in accordance with the incorporation of three SAM‐derived methyl groups. Cultivation of the strain in the presence of (*methyl*‐^2^H3)‐L‐methionine caused a mass shift of +8 for the molecular ion of **14**, which can only be due to the loss of one of the nine deuterium atoms contained in the three transferred methyl groups. Figure 6B illustrates the possible courses of the third methylation reaction catalyzed by the enzyme, which most probably starts from 4,5‐dimethyl‐IPP, which is the pyrophosphate form of the C_7_ compound **3**. After transfer of the third methyl group, the resulting carbocation can be directly quenched by deprotonation reactions at one the neighbored carbon atoms. The three respective products still contain all three transferred CH_3_ groups, which should result in mass shifts of +9 in a labelling experiment with (*methyl*‐^2^H3)‐L‐methionine. The already depicted abstraction of one of the nine protons, which were introduced as part of the three methyl groups is a reaction course that would be in accordance with the deuterium labelling experiment and is also shown in Figure 6B. This reaction path would include a 3,4 hydride shift.

The structural hypothesis for **14** of 3‐ethyl‐4‐methylpent‐4‐en‐1‐ol was also in line with the mass spectral fragmentation pattern (Figure 6C). After ionization the formation of the characteristic even fragment ion at *m*/*z* 84 can be explained by McLafferty rearrangement, a combined hydrogen rearrangement through a six‐membered transition state and subsequent α‐cleavage, with neutral loss of the enol of acetic aldehyde. Another α‐cleavage with loss of a Me group leads to *m*/*z* 69. For the structure of **14**, in the labelling experiment with (*methyl*‐^13^C)‐L‐methionine these fragment ions are expected at *m*/*z* 87 and 71, while they are expected at *m*/*z* 92 and 74 from (*methyl*‐^2^H_3_)‐L‐methionine, exactly as it is observed experimentally. To finally prove the hypothetical structure of **14**, the retention time and the mass spectrum of **14** with those of the reference compound 3‐ethyl‐4‐methylpent‐4‐en‐1‐ol were compared, showing their identity (Figure 6D and Figure S4N). The proposed 3,4 hydride shift in the course of the third methylation step catalyzed by the enzyme is only favored after double methylation at C4, because only then this hydride shift mediates between two tertiary carbocations, whereas single methylation at C4 does not allow for such a hydride shift that would produce a less stable secondary cation. This further rationalizes the enzymatic formation of **14** by a third methylation event.

For all GPP and IPP MTases characterized so far, quenching of the carbocation was demonstrated to proceed via deprotonation at one of the neighboring carbon atoms.[^15^, ^16^, ^17^, ^19^, ^20^, ^21^] Only in case of an FPP MTase from *Serratia plymuthica*, the carbocation formed after transfer of the methyl group to carbon atom 10 of FPP undergoes a series of methyl and hydride shifts, which finally lead to the release of the cyclized C_16_ prenyl pyrophosphate pre‐sodorifen.^[18]^ A functionally related enzyme, TleD from *Streptomyces blastmyceticus*, methylates the geranyl moiety of an intermediate in the teleocidin B biosynthesis pathway, which initiates a series of reactions also including a hydride shift.^[29]^ The IPP/DMAPP MTase from *M. humi* represents another enzyme, whose catalyzed reaction path involves not only methylation and deprotonation, but also a hydride shift step.

## Conclusion

Our study provides a detailed qualitative comparison of several different C_5_ prenyl pyrophosphate MTases. By the identification of four novel IPP MTases and several so far undescribed products, we were able to expand the knowledge about this enzyme group with strong biosynthetic potential. Our analyses demonstrate high deprotonation site selectivity, high stereoselectivity/enantioselectivity and high substrate selectivity for most of the enzymes investigated here. The ability to perform a second or even a third methylation seems to be considerably different for each catalyst. The enzyme humMT exhibits extraordinary features, as it not only accepts DMAPP besides IPP as substrate, but catalyzes the formation of a trimethylated prenyl pyrophosphate from IPP *in vivo*. Identification of the respective C_8_ terpene alcohol structure furthermore uncovered a 3,4 hydride shift in the course of the third methylation step. The broad diversity of these enzymes properties strongly expands the terpene building block repertoire and provides access to many terpene‐derived fine chemicals.

## Experimental Section

See the Supporting Information for full details. Including: Experimental descriptions. Supporting Table S1, Primers used in this study. Supporting Figure S1, SDS‐PAGE analysis of purified enzymes. Supporting Figure S2, Graphic representation of genes encoding putative SAM‐dependent prenyl pyrophosphate MTases and flanking genes. Supporting Figure S3, GC chromatograms (TIC) of the reference compounds which could be assigned to MTase products. Supporting Figure S4 A–N, Mass spectra of all compounds detected in in vivo or in vivo experiments and of respective reference compounds. Supporting Figure S5, Total ion chromatograms of DMAPP conversion product and reference compounds. Supporting Figure S6. Synthesis of A) (2*S*)‐2‐methyl‐IPP and B) (2*R*)‐2‐methyl‐IPP. Supporting Figures S7–S28, NMR analysis.

## Conflict of interest

The authors declare no conflict of interest.

1

## Supporting information

As a service to our authors and readers, this journal provides supporting information supplied by the authors. Such materials are peer reviewed and may be re‐organized for online delivery, but are not copy‐edited or typeset. Technical support issues arising from supporting information (other than missing files) should be addressed to the authors.

Supporting Information

## Data Availability

The data that support the findings of this study are available in the supplementary material of this article.

## References

[1] J. Buckingham, *Dictionary of Natural Products, version 26.1*.

[2] O. Wallach , Liebigs Ann. 1885, 227, 277.

[3] L. Ruzicka , Experientia 1953, 9, 357–367. 13116962 10.1007/BF02167631

[4] J. Jiang , D. E. Cane , J. Am. Chem. Soc. 2008, 130, 428–429. 18095692 10.1021/ja077792i PMC2533842

[5] K. H. Dahm , H. Röller , Life Sci. 1970, 9, 1397–1400. 10.1016/0024-3205(70)90099-8 5493010

[6] A. S. Meyer , H. A. Schneiderman , E. Hanzmann , J. H. Ko , Proc. Natl. Acad. Sci. USA 1968, 60, 853–860. 16591663 10.1073/pnas.60.3.853 PMC225130

[7] F. C. Baker , D. A. Schooley , Biochim. Biophys. Acta 1981, 664, 356–372. 6166327 10.1016/0005-2760(81)90058-8

[8] R. C. Jennings , K. J. Judy , D. A. Schooley , M. Sharon Hall , J. B. Siddall , Life Sci. 1975, 16, 1033–1039. 1134181 10.1016/0024-3205(75)90187-3

[9] M. G. Peter , K. H. Dahm , Helv. Chim. Acta 1975, 58, 1037–1048. 1158736 10.1002/hlca.19750580407

[10] H. Rodé-Gowal , S. Abbott , D. Meyer , H. Röller , K. H. Dahm , Z. Naturforsch. C 1975, 30, 392–397.

[11] J. G. Hamilton , G. W. Dawson , J. A. Pickett , J. Chem. Ecol. 1996, 22, 1477–1491. 24226250 10.1007/BF02027726

[12] J. G. Hamilton , G. W. Dawson , J. A. Pickett , J. Chem. Ecol. 1996, 22, 2331–2340. 24227307 10.1007/BF02029550

[13] F. J. Ritter , I. E. M. Brüggemann-Rotgans , P. Verwiel , C. J. Persoons , E. Talman , Tetrahedron Lett. 1977, 18, 2617–2618.

[14] B. W. Staddon , A. Abdollahi , J. Parry , M. Rossiter , D. W. Knight , J. Chem. Ecol. 1994, 20, 2721–2731. 24241843 10.1007/BF02036203

[15] J. S. Dickschat , T. Nawrath , V. Thiel , B. Kunze , R. Muller , S. Schulz , Angew. Chem. Int. Ed. 2007, 46, 8287–8290; 10.1002/anie.200702496 17899580

[16] M. Komatsu , M. Tsuda , S. Omura , H. Oikawa , H. Ikeda , Proc. Natl. Acad. Sci. USA 2008, 105, 7422–7427. 18492804 10.1073/pnas.0802312105 PMC2387273

[17] C. M. Wang , D. E. Cane , J. Am. Chem. Soc. 2008, 130, 8908–8909. 18563898 10.1021/ja803639g PMC3023297

[18] S. von Reuss , D. Domik , M. C. Lemfack , N. Magnus , M. Kai , T. Weise , B. Piechulla , J. Am. Chem. Soc. 2018, 140, 11855–11862. 30133268 10.1021/jacs.8b08510

[19] V. Radhika , N. Ueda , Y. Tsuboi , M. Kojima , J. Kikuchi , T. Kudo , H. Sakakibara , Plant Physiol. 2015, 169, 1118–1126. 26251309 10.1104/pp.15.00787 PMC4587462

[20] T. Ozaki , S. S. Shinde , L. Gao , R. Okuizumi , C. Liu , Y. Ogasawara , X. Lei , T. Dairi , A. Minami , H. Oikawa , Angew. Chem. Int. Ed. 2018, 57, 6629–6632; 10.1002/anie.201802116 29603559

[21] L. Drummond , M. J. Kschowak , J. Breitenbach , H. Wolff , Y. M. Shi , J. Schrader , H. B. Bode , G. Sandmann , M. Buchhaupt , ACS Synth. Biol. 2019, 8, 1303–1313. 31059642 10.1021/acssynbio.8b00525

[22] O. Ariyawutthiphan , T. Ose , A. Minami , S. Sinde , M. Tsuda , Y.-G. Gao , M. Yao , H. Oikawa , I. Tanaka , Acta Crystallogr. Sect. D 2012, 68, 1558–1569. 23090405 10.1107/S0907444912038486

[23] C. Ignea , M. Pontini , M. S. Motawia , M. E. Maffei , A. M. Makris , S. C. Kampranis , Nat. Chem. Biol. 2018, 14, 1090–1098. 30429605 10.1038/s41589-018-0166-5

[24] M. J. Kschowak , H. Wortmann , J. S. Dickschat , J. Schrader , M. Buchhaupt , PLoS One 2018, 13, e0196082. 29672609 10.1371/journal.pone.0196082 PMC5908152

[25] J. Couillaud , K. Duquesne , G. Iacazio , ChemBioChem 2021, 23, e202100642. 34905641 10.1002/cbic.202100642

[26] V. Harms , A. Kirschning , J. S. Dickschat , Nat. Prod. Rep. 2020, 37, 1080–1097. 32068211 10.1039/c9np00055k

[27] A. A. Malico , M. A. Calzini , A. K. Gayen , G. J. Williams , J. Ind. Microbiol. Biotechnol. 2020, 47, 675–702. 32880770 10.1007/s10295-020-02306-3 PMC7666032

[28] C. Akil, Master's Thesis, *Izmir Institute of Technology* **2014**.

[29] T. Awakawa , L. Zhang , T. Wakimoto , S. Hoshino , T. Mori , T. Ito , J. Ishikawa , M. E. Tanner , I. Abe , J. Am. Chem. Soc. 2014, 136, 9910–9913. 24992358 10.1021/ja505224r

