# Supplementary material for: High Versatility of IPP and DMAPP Methyltransferases Enables Synthesis of C6, C7 and C8 Terpenoid Building Blocks
Source: Chembiochem. 2022 Jun 7;23(14):e202200091. doi: 10.1002/cbic.202200091 (PMC9401056; doi:10.1002/cbic.202200091)
Supplement: Supplementary file 1 — Supporting Information [file CBIC-23-0-s001.pdf]

ChemBioChem

Supporting Information

High Versatility of IPP and DMAPP Methyltransferases Enables Synthesis of C_6, C_7 and C_8 Terpenoid Building Blocks

Laura Drummond[+], Parab J. Haque[+], Binbin Gu, Julia S. Jung, Hendrik Schewe, Jeroen S. Dickschat, and Markus Buchhaupt*

Table of Contents

S1 Chemicals, Media, and Microorganisms

S2 Vector construction

S3 Enzyme expression and purification

S4 *In vitro* MTase reactions

S5 *De novo* terpenoid production in *E. coli* strains containing the mevalonate pathway and one of the MTases

S6 HS-SPME-GC/MS analysis

Supplementary Table 1 Primers used in this study

Supplementary Figure S1. SDS-PAGE analysis of purified enzymes

Supplementary Figure S2. Graphic representation of genes encoding putative SAM-dependent prenyl pyrophosphate MTases and flanking genes

Supplementary Figure S3. GC chromatograms (TIC) of the reference compounds which could be assigned to MTase products.

Supplementary Figure S4. Mass spectra of all compounds detected in *in vivo* or *in vivo* experiments and of respective reference compounds

Supplementary Figure S5. Total ion chromatograms of DMAPP conversion product and reference compounds

Supplementary Figure S6. Synthesis of A) (2*S*)-2-methyl-IPP and B) (2*R*)-2-methyl-IPP.

S7 Synthesis of (*R*)- and (*S*)-methyl 3-(*tert*-butyldiphenylsilyloxy)-2-methylpropanoate (S2)

S8 Synthesis of (*R*)- and (*S*)-3-(*tert*-butyldiphenylsilyloxy)-*N*-methoxy-*N*,2-dimethylpropanamide (S3)

S9 Synthesis of (*R*)- and (*S*)-4-(*tert*-butyldiphenylsilyloxy)-3-methylbutan-2-one (S4)

S10 Synthesis of (*S*)- and (*R*)-*tert*-butyl((2,3-dimethylbut-3-en-1-yl)oxy)diphenylsilane (S5)

S11 Synthesis of (*S*)- and (*R*)-2,3-dimethylbut-3-en-1-ol (S6)

S12 Synthesis of (*S*)- and (*R*)-2,3-dimethylbut-3-en-1-yl 4-methylbenzenesulfonate (S7)

S13 Synthesis of (*S*)- and (*R*)-2,3-dimethylbut-3-en-1-yl diphosphate (S8)

Supplementary Figures S7 – S28 NMR analyses

S1: Chemicals, Media, and Microorganisms
E. coli strain DH5α (New England Biolabs) was used for plasmid construction. *E. coli* BL21 (DE3) (Novagen) was used for His-tag protein expression. *E. coli* MG1655 (DE3) Δ*recA* Δ*endA* Δ*tnaA* was used for *de novo* terpenoid production experiments. It was constructed from *E. coli* MG1655 (DE3) Δ*recA* Δ*endA* (Addgene #37854) with the Quick and Easy *E. coli* Gene Deletion Kit (GeneBridges, Heidelberg) and the oligonucleotides DtnaA-1 and DtnaA-2. *E. coli* strains were grown in LB medium or TB medium containing the appropriate antibiotics (ampicillin at 50 µg/L, kanamycin at 50 µg/L, gentamycin 10 µg/L), at 37°C and 180 rpm. Solid medium was prepared by the addition of 1.5 % agar-agar (m/v).

Solid-Phase Micro-Extraction (SPME) fibers, DTT, acid phosphatase, IPP, S-Adenosylmethionine (SAM), RNAse, DNAse and protease inhibitor were purchased from Sigma-Aldrich. Reference compounds purchased from the company Enamine Ltd (Riga, Latvia) are named here with corresponding product numbers: (*E*)-4-methyl-prenol (Z3488308004), (*Z*)-4-methyl-prenol (Z3773228680), 2,4-dimethyl-isoprenol (Z1896357284), 2,5-dimethyl-isoprenol (Z1896382803), 4,4-dimethyl-prenol (FCH2520219), 4,5-dimethyl-prenol (EN300-2524336), 4,5-dimethyl-isoprenol (Z3681219426) and 5,5-dimethyl-isoprenol (EN300-123672) were synthesized by Enamine Ltd (Riga, Latvia). For all C_7 compounds, mixtures of diastereomers or enantiomers were used. 3-ethyl-4-methylpent-4-en-1-ol was synthesized by Genosynth (Berlin, Germany). All standard compounds had purities of at least 95% and had their identities confirmed by NMR and GC−MS by the synthesizing companies.

S2: Vector construction
Construction of pET-28a(+) vectors was carried out by BioCat GmbH (Heidelberg, Germany) or via Gibson assembly. The following sequences were used in codon-optimized or natural forms to construct the respective vectors: pPJH-1 (WP_091072690 from *M. humi*, humMT), pPJH-2 (WP_032365004 from *R. fascians*, fasMT), pPJH-3 (WP_030281021 from *S. catenulae*, catMT), pPJH-4 (WP_076843458 from *Frankia* sp., fraMT), pPJH-5 (WP_005164808 from *A. azurea*, azuMT), pPJH-6 (BAF98640 from *S. argenteolus*, argMT). Construction of pMK-24 containing the monMT-encoding gene has been described.[1] The methyltransferase-encoding genes from the extracted genomic DNA of *M. humi* DSM 45647, *R. fascians*-1 DSM 20699 and *A. azurea* DSM 43854 were amplified via PCR using gene-specific primers (Supplementary Table 1) to form the vectors pPJH-1, pPJH-2 and pPJH-6. Successful construction of the vectors was validated by Sanger sequencing. Vectors pPJH-3, pPJH-4, pPJH-5, and pPJH-7 were synthesized by BioCat GmbH via standard restriction-cloning. Vector pLD-03 was used to provide high IPP and DMAPP levels and its construction has been described.[1]

S3: Enzyme expression and purification
E. coli strain BL21(DE3) was transformed with one of the pET-28a(+) derivatives containing one of the MTase-encoding genes. LB pre-cultures were used to inoculate main cultures with 1 L TB medium containing ampicillin (50 µg/ml) or kanamycin (50 µg/ml). After cultivation for 2 h at 37 °C (OD_{600} of 0.8), IPTG at 100 µM was added for induction. After cultivation at 18 °C, 110 rpm overnight, cells were harvested by centrifugation for 20 minutes at 4000 g and 4°C then re-suspended in 15 ml lysis buffer. Ultrasound sonication was performed on ice with 20 % amplitude (0.5 seconds pulse, 1 second pause, 4 minutes pulse time), followed by digestion with RNAse (10 µg/ml) and DNAse (5 µg/ml) on ice for 10 min. After centrifugation for 20 minutes at 4000 g and 4 °C, enzyme purification took place using His-Pur Ni-NTA Spin

Columns (Thermo Fischer Scientific) according to the manufacturer's recommendations using the following buffers. Assay buffer (pH 6.7) consisted of 20 mM Tris-HCl, 100 mM NaCl, 15 mM $MgCl_2$, and 5 mM DTT in ultrapure water. Lysis, wash and elution buffers had pH of 8 and consisted of 50 mM Tris-HCl, 500 mM NaCl, 5 mM DTT with increasing concentrations of imidazole through each step: 10 mM (lysis), 30 mM (wash) and 250 mM (elution). Purified proteins were analyzed via SDS-PAGE using 15 µg of protein (Figure S1). Glycerol, Tris-HCl, NaCl, imidazole, ampicillin and kanamycin were purchased from Carl Roth GmbH.

S4: *In vitro* MTase reactions

Purified protein at 25 µM final concentration was added to 1 mL assay buffer (20 mM Tris-HCl, 100 mM NaCl, 15 mM $MgCl_2$, and 5 mM DTT, pH 6.7 in ultrapure water) containing 120 µM SAM and 60 µM of IPP, DMAPP, GPP or FPP. The enzymatic reaction was incubated at 30 °C overnight. Then 1 ml phosphatase solution (acid phosphatase 7.5 mg/ml in 0.1 M sodium acetate, pH 5.0) was added and the reaction was incubated at 30 °C for 2 h. Following the reaction, volatiles in the headspace were extracted with a SPME fibre for 20 minutes.

S5: *De novo* terpenoid production in *E. coli* strains containing the mevalonate pathway and one of the MTases

The production strain MG1655 harbored the plasmid pMK-16 for provision of high IPP and DMAPP levels and pET-28a derivatives containing the sequence of a MTase. Pre-cultures in reaction tubes containing 5 mL LB medium with appropriate antibiotics were incubated overnight at 37°C and 180 rpm. Main cultures with 15 mL TB medium and appropriate antibiotics in 100 ml baffled shake flasks were inoculated from pre-culture to an OD_{600} of 0.1. After cultivation at 37°C to an OD_{600} value of 1, gene expression was induced with isopropyl-β-D-1-thiogalactopyranoside (IPTG, 100 µM). Induced cultures had terpenoids extracted during the following 24 hours of cultivation at 30°C and 180 rpm with a magnetic adsorbent stir bar (Twister® , Gerstel). The twister was attached to the shake flask with the help of a magnet fixated to the external part of the flask, thus preventing the twister from being loose on the culture medium. If indicated, unlabeled L-methionine, ^{13}C-methyl-L-methionine or (methyl-d_3)-L-methionine were supplemented (c_{final} = 3 g/L) to the pre- and main cultures.

S6: HS-SPME-GC/MS analysis

Volatile compounds in the headspace of *in vitro* assays and cultures were analyzed by extraction with an 85 µm stableflex SPME fiber composed of PDMS and Carboxen. The SPME fibre placed in a fibre holder (Gerstel) and attached to a stainless steel plunger sheathed by a protective needle (Supelco) was exposed in the headspace of each assay and culture and then inserted into the injection port of a GC-MS-QP2010 (Shimadzu) containing a DB-5 (5 %-phenyl)-methylpolysiloxane column with 30 m length and 0.25 mm thickness. Measurements were conducted as follows: helium as carrier gas, splitless injections at 250 °C, 1 minute sampling time, and column flow of 1.1 mL/min. The column temperature was programmed to start with 40 °C for 1.5 minute then rising in steps of 10°C/min until 250 °C followed by rising in 20 °C/min steps up to 300°C. Compounds were identified via comparison of mass spectra and retention indices (RI) to the ones of reference substances or mass spectra of the NIST mass spectral library (v14).

For chiral analysis of the product of DMAPP methylation by humMT, an Astec CHIRALDEX B-DA cyclodextrin stationary phase column connected to an inactivated guard column was employed in a GC-MS QP2010 (Shimadzu) system. An SPME fibre was used for extraction of volatiles on the headspace of the *in vitro* assays with a final step of phosphatase reaction, as

described in the paragraph above. To elucidate product chirality, the two enantiomers (2*R*)-2-methyl-IPP and (2*S*)-2-methyl-IPP (synthesis described on Supplementary Information S3) were analyzed separately, by adding each synthetic prenyl pyrophosphate at a final concentration of 6 µM to a solution containing 7,5 mg/mL acid phosphatase. After incubation for 30 min at 40 °C, an SPME fibre was used to explore the headspace of the assay for 10 min at 40 °C. The retention times of both enantiomers were then compared to the one from the product generated by enzymatic assay. SPME-GC-MS measurements were conducted as follows: helium as carrier gas, splitless injections at 260 °C, 1 minute sampling time, and column flow of 0.84 mL/min. The column temperature was programmed to start with 40 °C for 2 minutes then rising in steps of 5 °C/min until 120 °C, hold for 40 minutes followed by rising in 10 °C/min steps up to 180 °C, hold for 5 minutes. Temperature was then decreased at a rate of -10 °C/min until 130 °C, hold for 1 minute and then decreased at a rate of -5 °C until 40 °C. Compounds were identified via comparison of mass spectra and retention indices (RI) to the ones of reference substances.

Supplementary Table 1

Name	Sequence	Purpose
pPJH-1A	GCTAGCATGACTGGTGGACAGCAAATGGGTATGTCGATC GATCTCGGC	Construction of pPJH-1
pPJH-1B	CGGATCTCAGTGGTGGTGGTGGTGGTGGTCGGTGG GTTCGAC	Construction of pPJH-1
pPJH-2A	GCTAGCATGACTGGTGGACAGCAAATGGGTATGCCGAAC CTCGACGTG	Construction of pPJH-2
pPJH-2B	CAGTGGTGGTGGTGGTGGTGCAGACGCTCGCACGC	Construction of pPJH-2
pPJH-4A	CATGACTGGTGGACAGCAAATGGGTATGAATTCCAACAA CAGCTCC	Construction of pPJH-4
pPJH-4B	GATCTCAGTGGTGGTGGTGGTGGTGGCGATCGGTGTTCGG	Construction of pPJH-4
DtnaA-1	TGTAATATTCACAGGGATCACTGTAATTAAAATAAATGAAG GATTATGTAAATTAACCCTCACTAAAGGGCGG	Deletion of *tnaA*
DtnaA-2	TGTAGGGTAAGAGAGTGGCTAACATCCTTATAGCCACTCTG TAGTATTA ATAATACGACTCACTATAGGGCTC	Deletion of *tnaA*

Supplementary Figure S1 SDS-PAGE analysis of purified enzymes. 15 µg of purified protein was used per lane. Gels were stained with Coomassie Brilliant Blue. Page ruler Prestained protein ladder, 10 – 180 kDa (Thermo Fischer Scientific) was used as protein size reference. Lanes were spliced to show the purified protein sample beside the respective protein ladder from the same gel, which is indicated by the black lines.

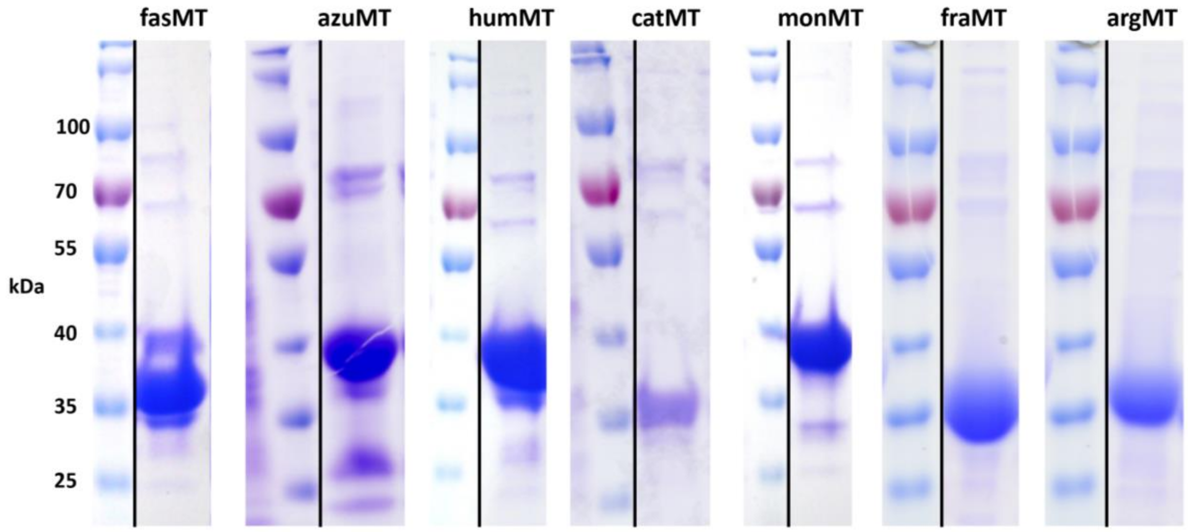

Supplementary Figure S2 Graphic representation of genes encoding putative SAM-dependent prenyl pyrophosphate MTases and flanking genes. The red arrows represent the putative prenyl pyrophosphate MTase-encoding genes selected for further analysis. Putative functions of the proteins encoded upstream and downstream of the MTase gene were identified by the search for specific protein domains and by the search for similar protein sequences with known function.

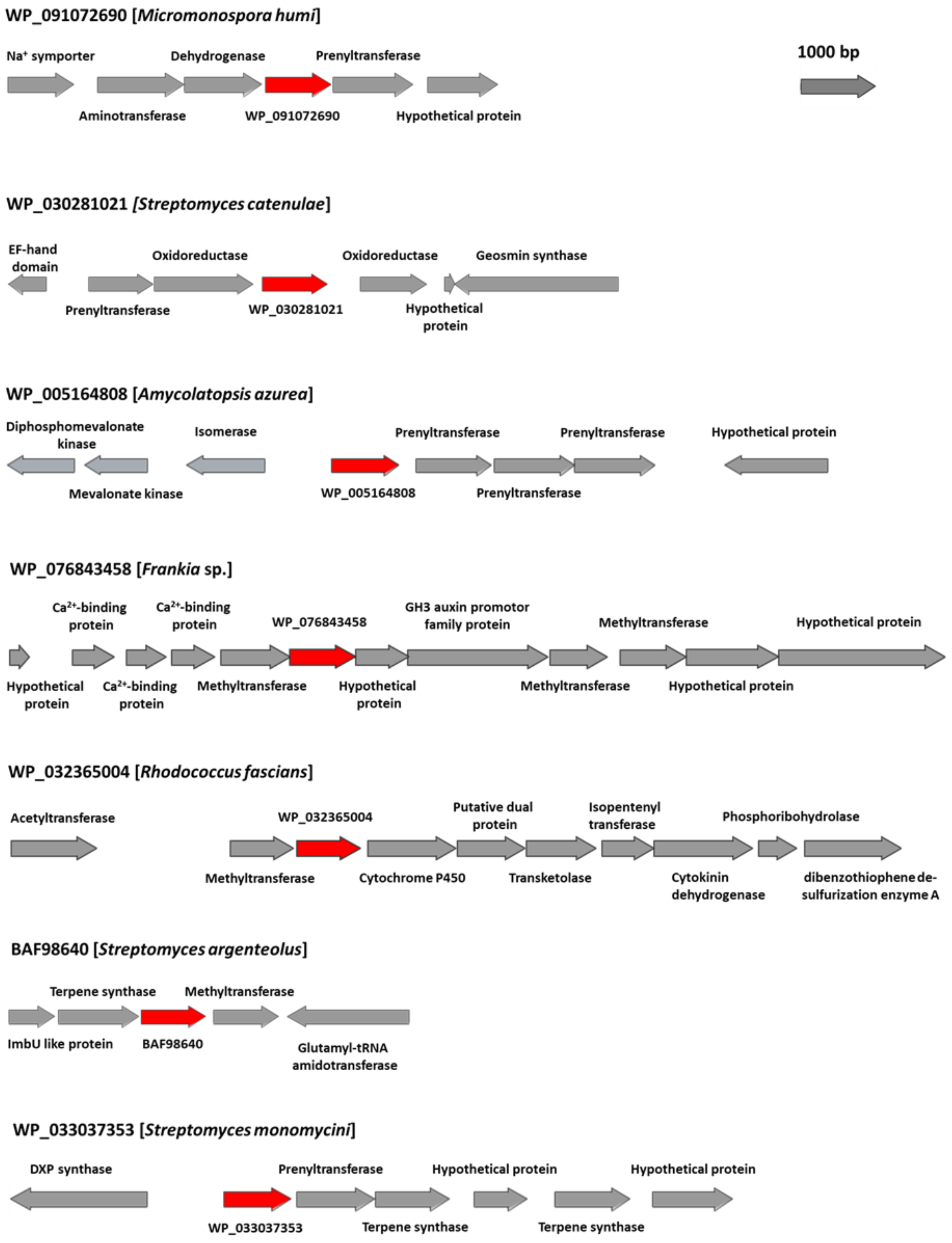

Supplementary Figure S3. GC chromatograms (TIC) of the reference compounds which could be assigned to MTase products. Red dots in the respective structures indicate the positions of the additional methyl groups. In case of 4,5-dimethyl-isoprenol it is possible to see two peaks corresponding to the two isomers present in the mixture, although it is unclear, which peak represents which isomer.

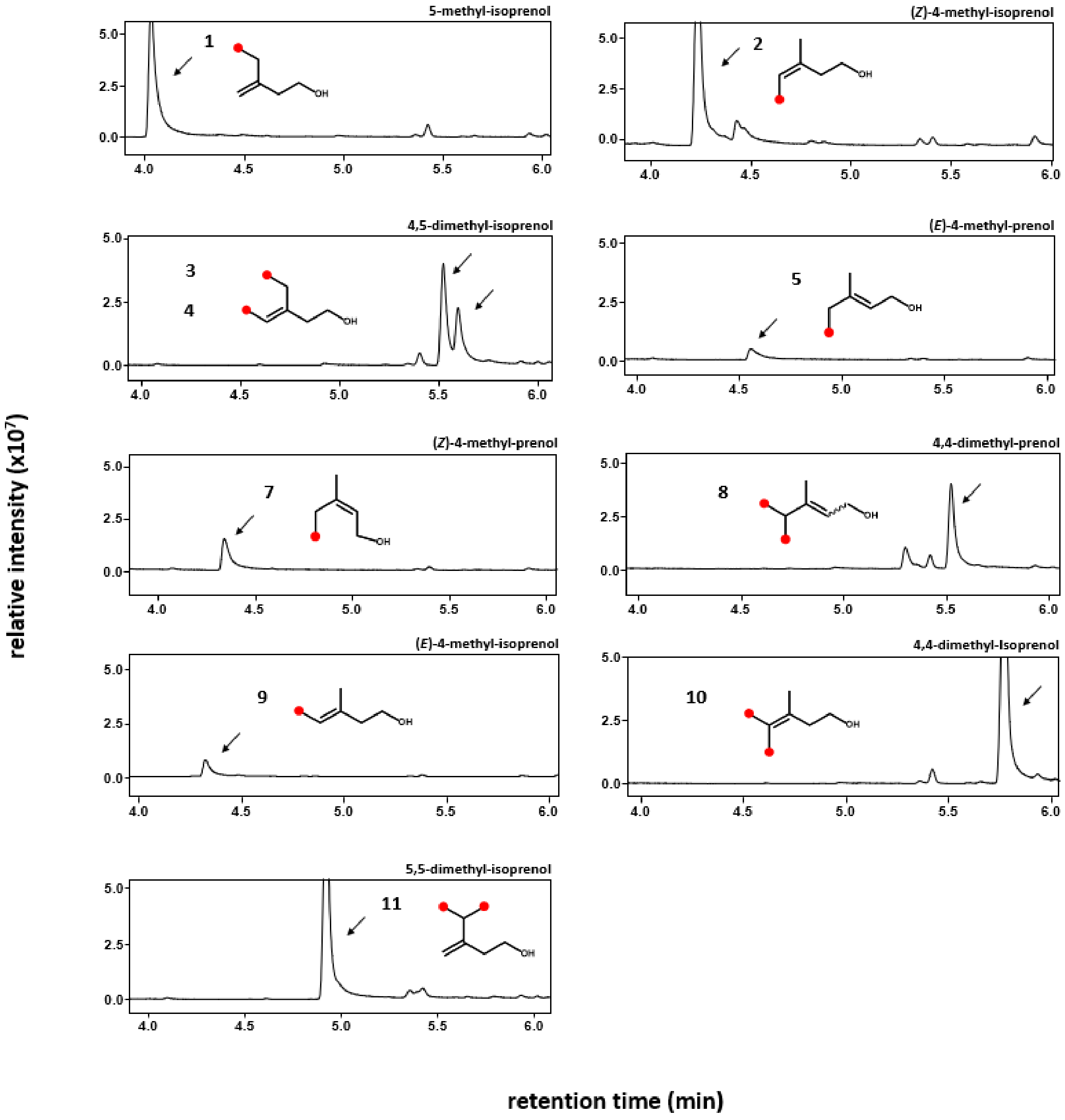

Supplementary Figure S4. Mass spectra of all compounds detected in *in vivo* or *in vitro* experiments and of respective reference compounds. In all cases, in which an enzymatically or microbially synthesized compound could be clearly assigned to one of the reference compounds, both mass spectra are shown as comparison. In the case of unknown C_7 compound **6** only the mass spectrum of detected compound is shown.

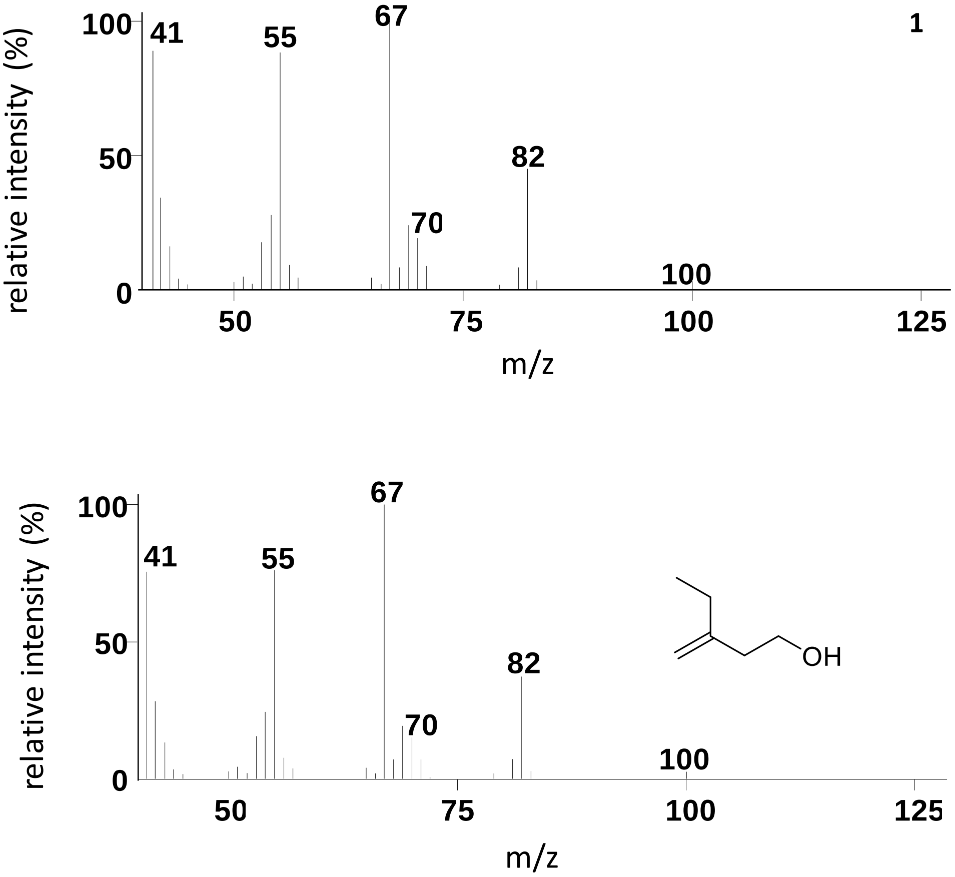

S4A. Comparison of MS of **1** detected (upper) and 5-methyl-isoprenol reference compound (lower)

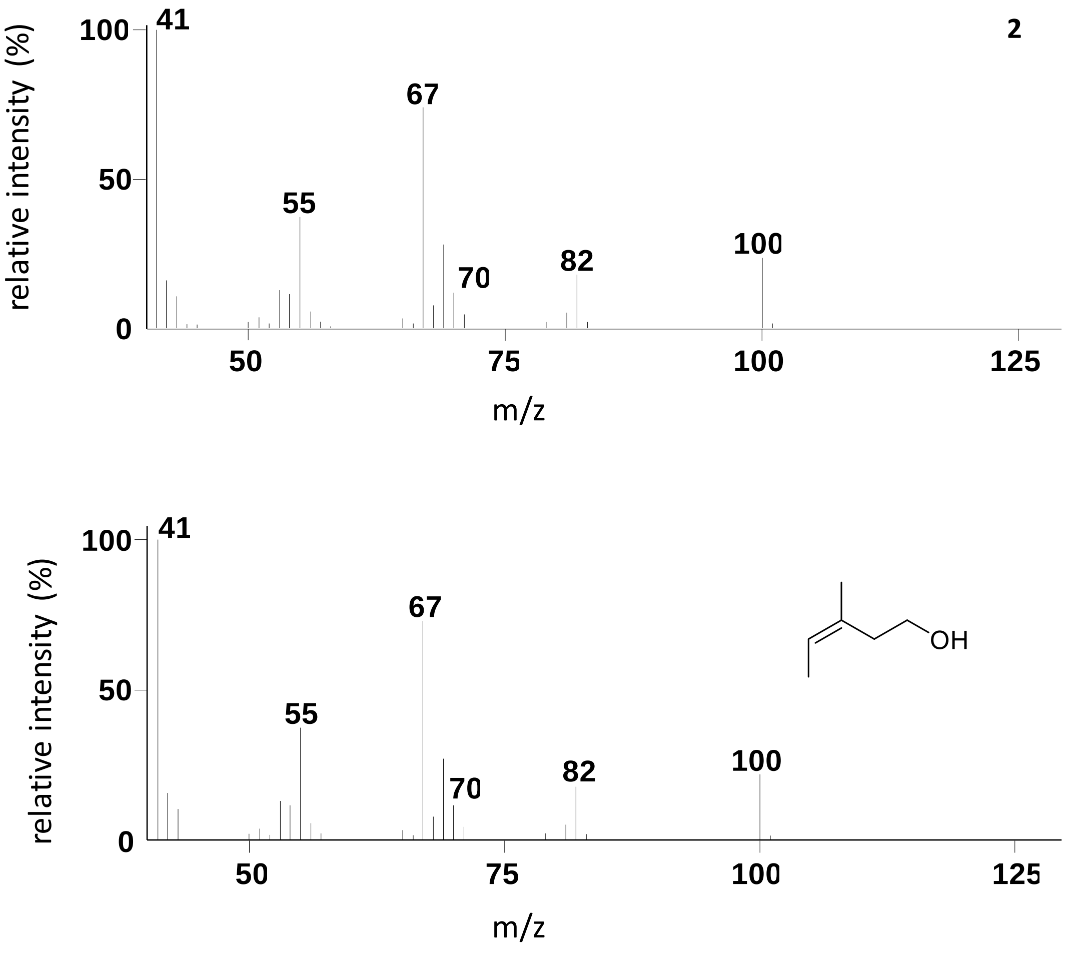

S4B. Comparison of MS of **2** detected (upper) and (*Z*)-4-methyl-isoprenol (lower).

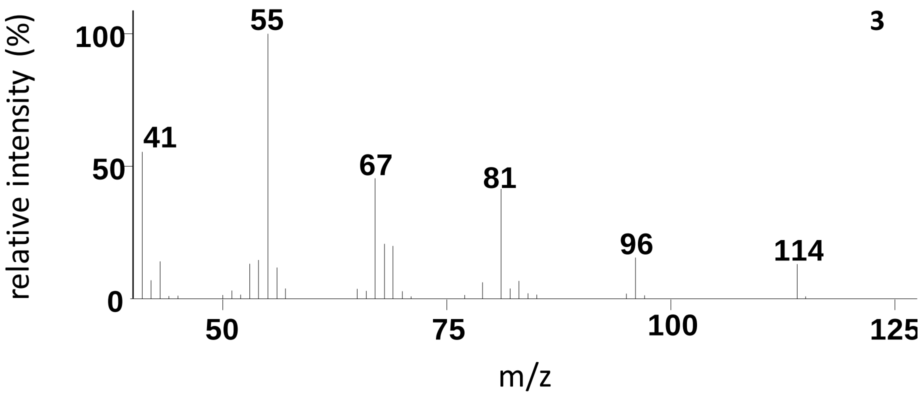

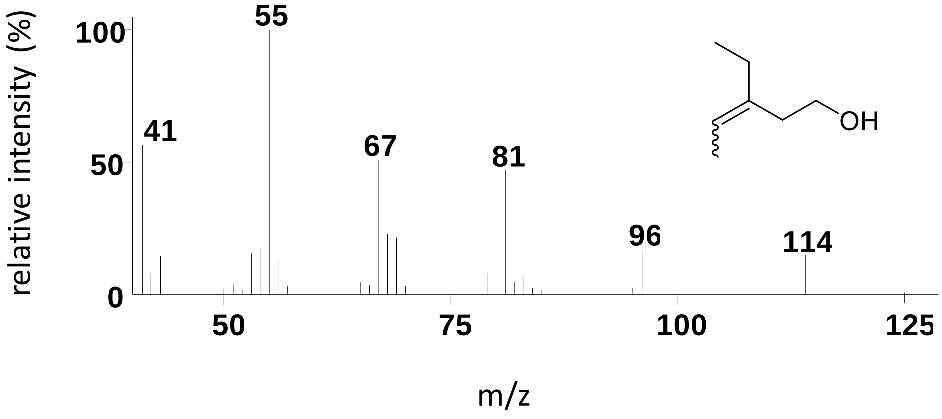

S4C. Comparison of MS of **3** detected (upper) and one of the stereoisomers of 4,5-dimethyl-isoprenol (lower).

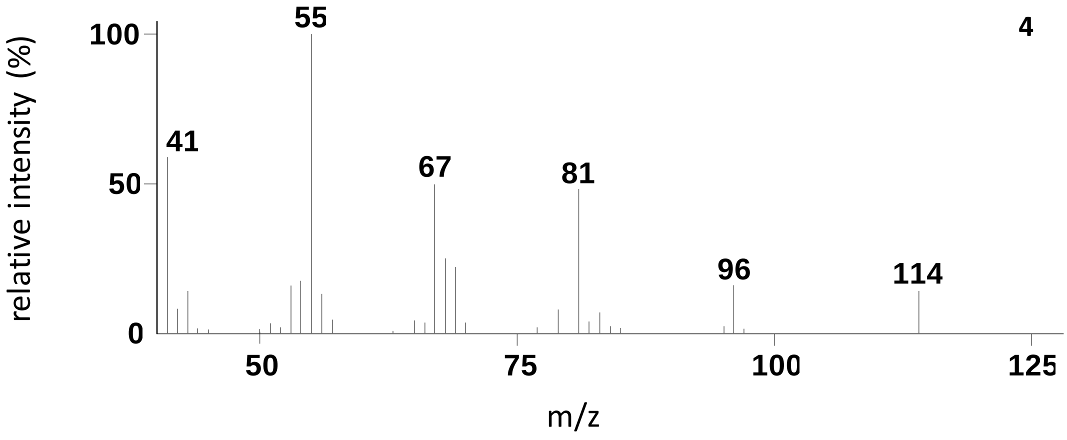

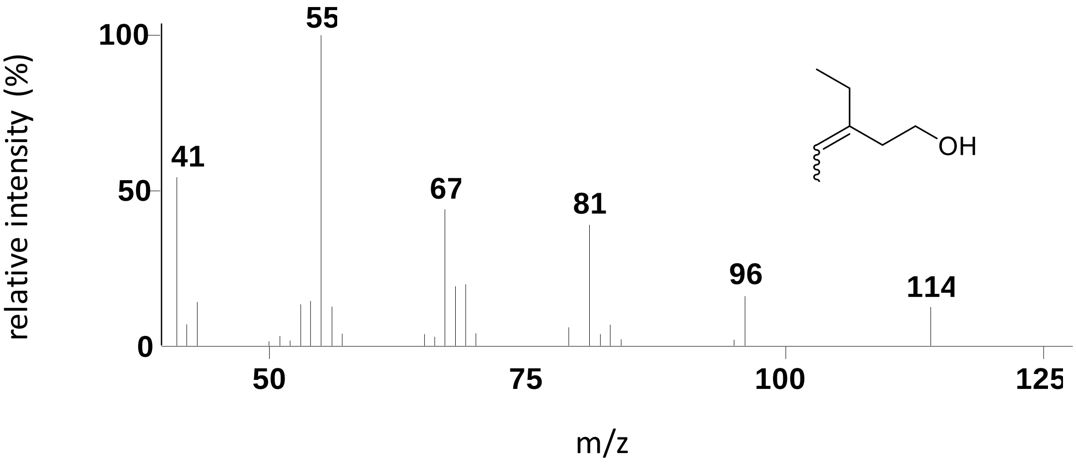

S4D. Comparison of MS of **4** detected (upper) and one of the stereoisomers of 4,5-dimethyl-isoprenol (lower).

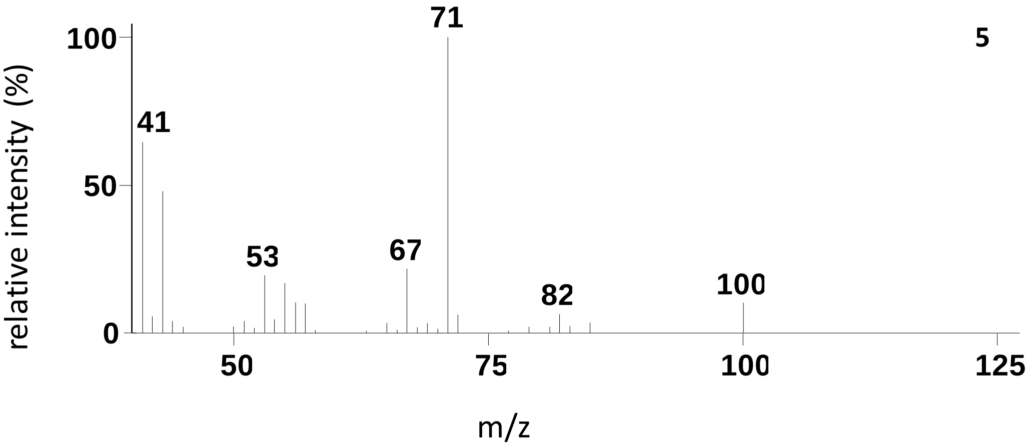

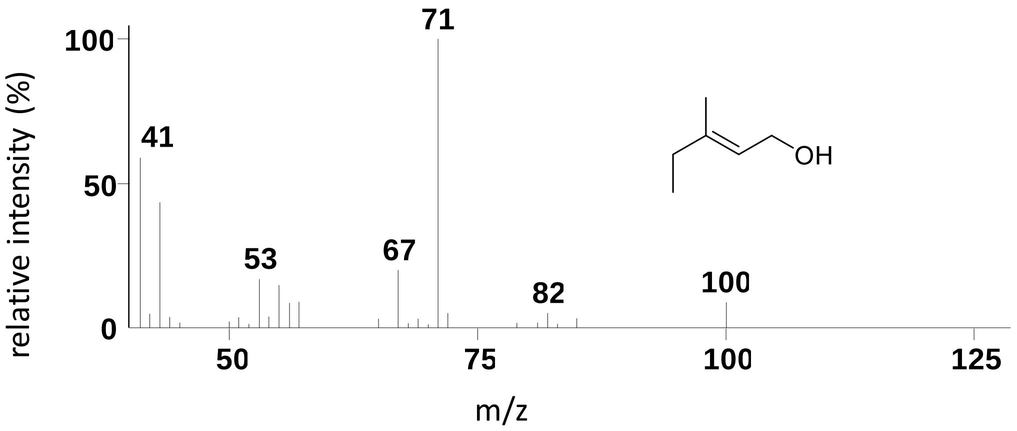

S4E. Comparison of MS of **5** detected (upper) and (*E*)-4-methyl-prenol (lower).

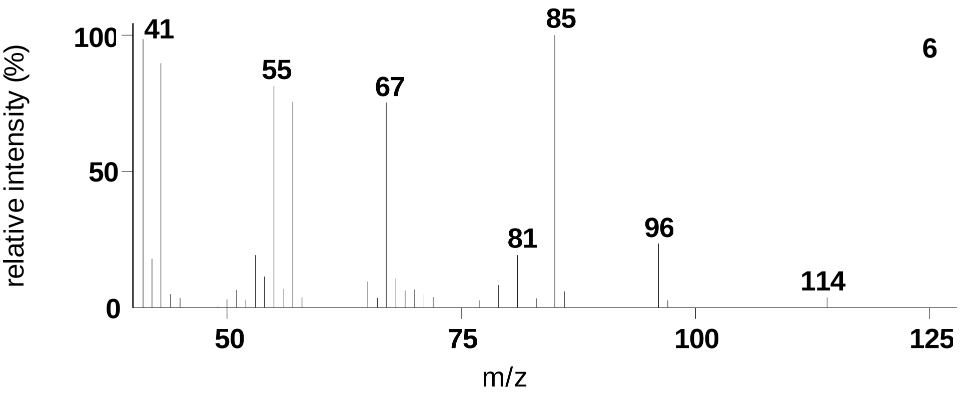

S4F. Mass spectrum of unknown C_7 compound **6** detected.

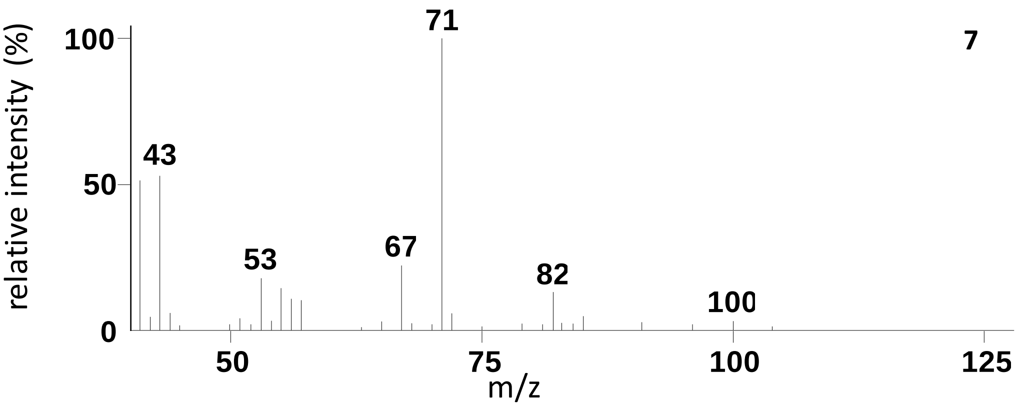

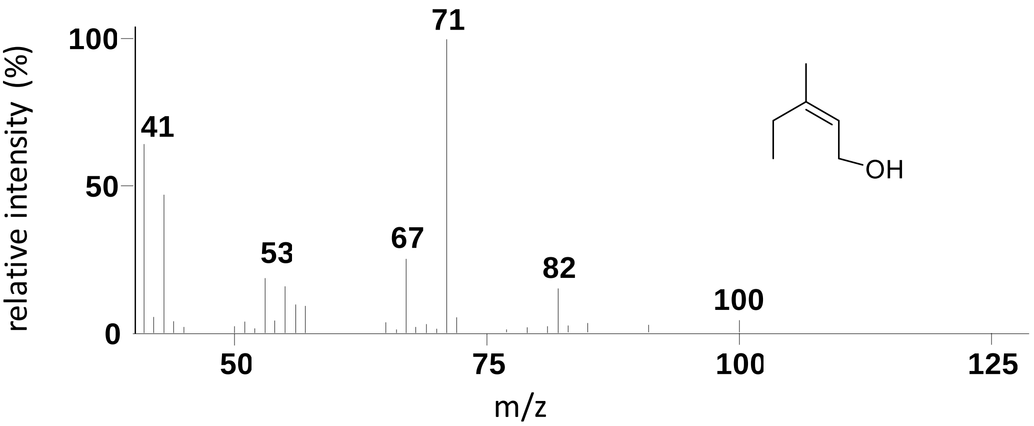

S4G. Comparison of MS of **7** detected (upper) and (*Z*)-4-methyl-prenol (lower).

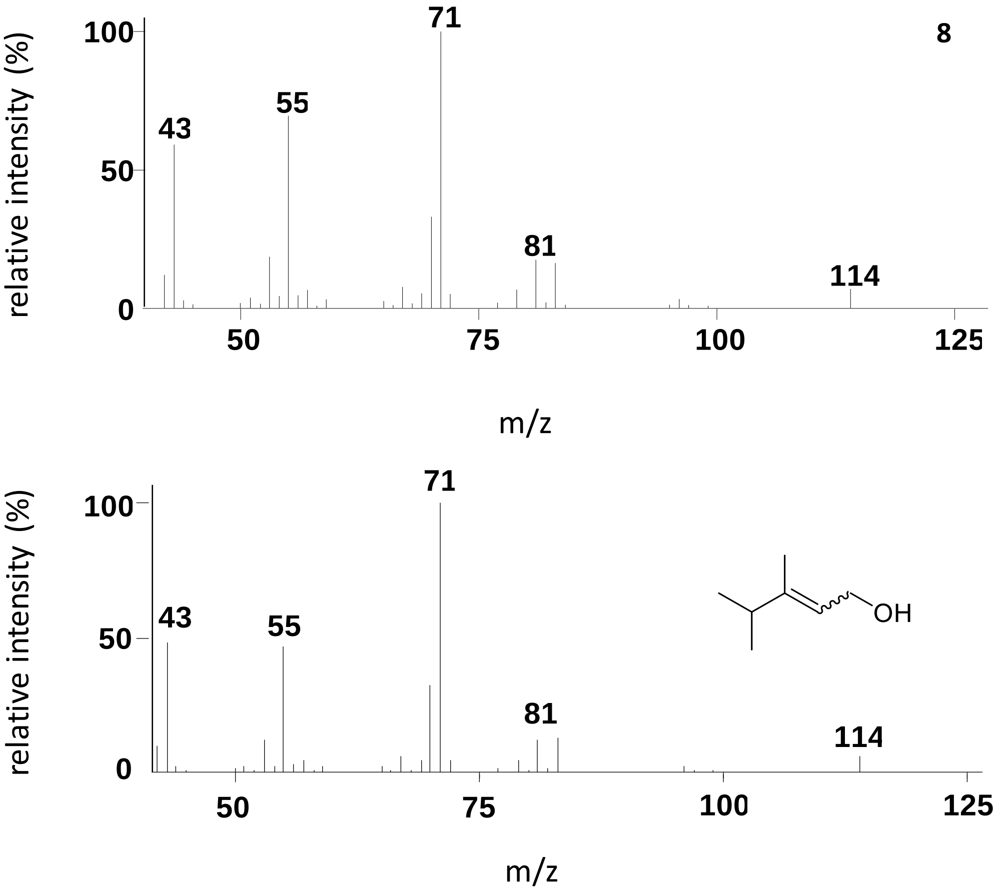

S4H. Comparison of MS of **8** detected (upper) and 4,4-dimethyl-prenol (lower).

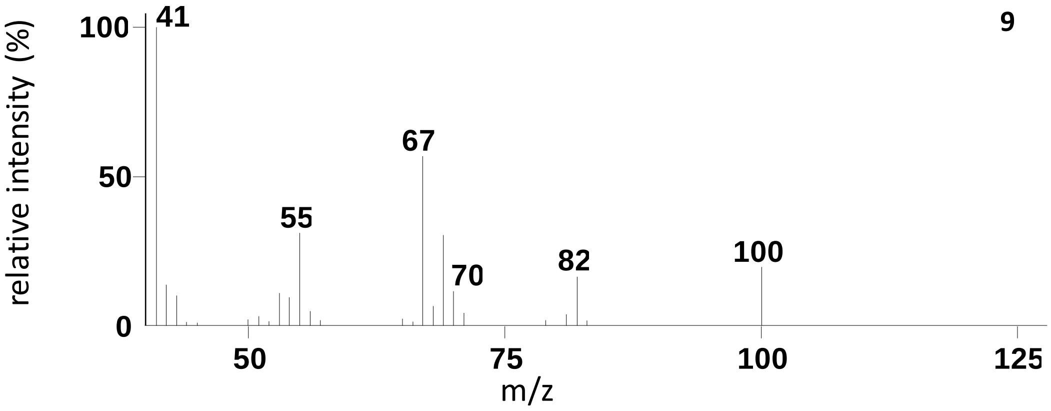

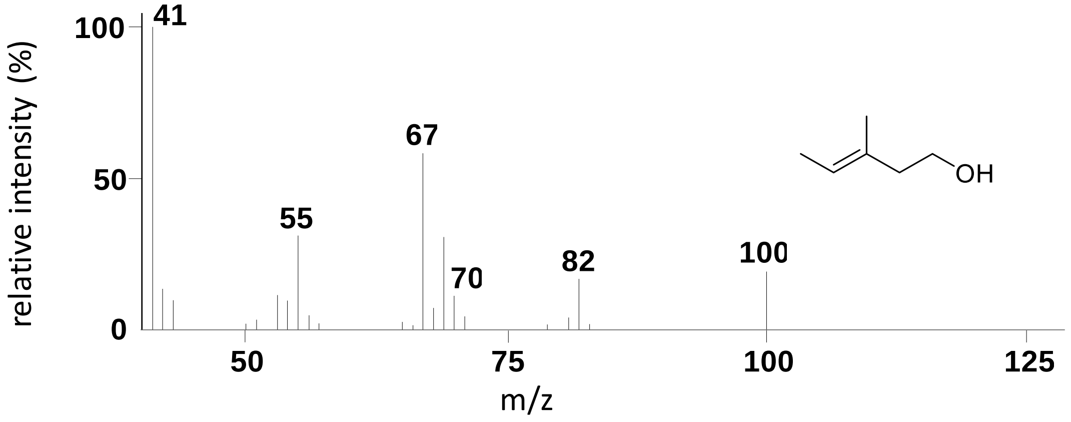

S4I. Comparison of MS of **9** detected (upper) and (*E*)-4-methyl-isoprenol (lower).

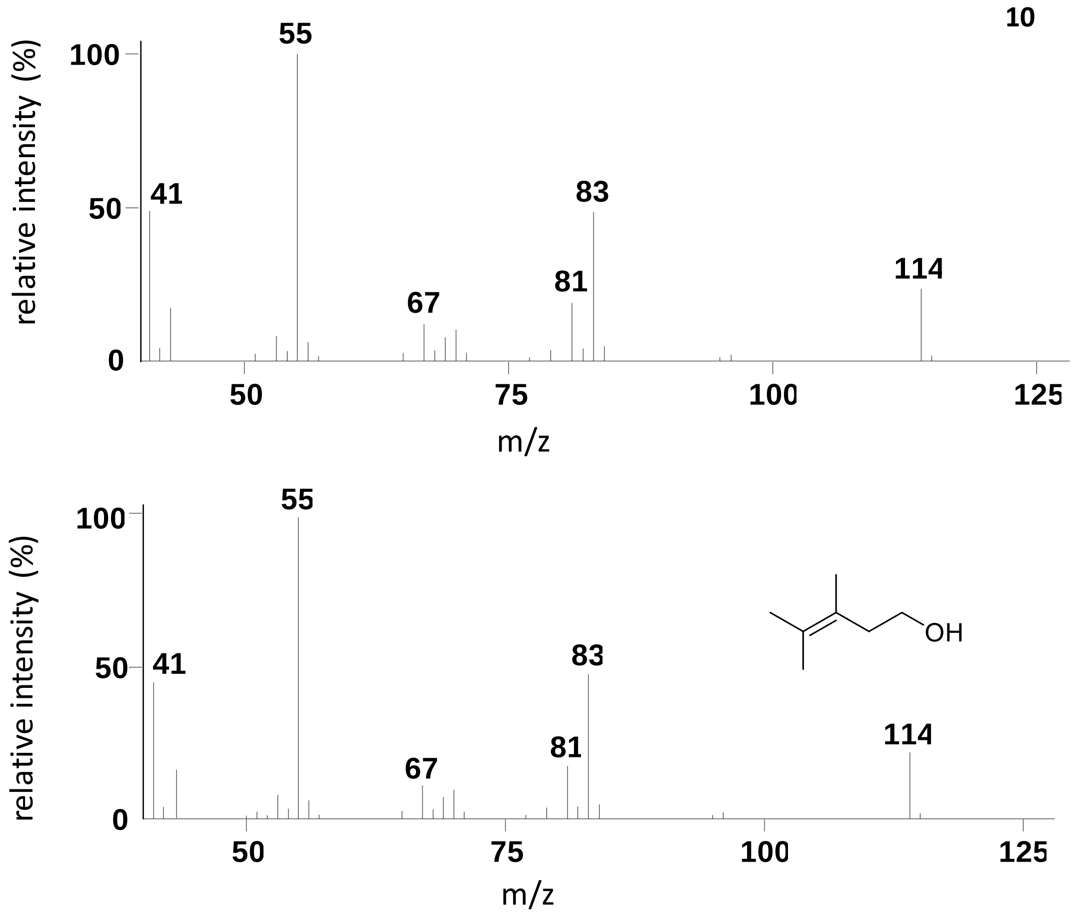

S4J. Comparison of MS of **10** detected (upper) and 4,4-dimethyl-isoprenol (lower).

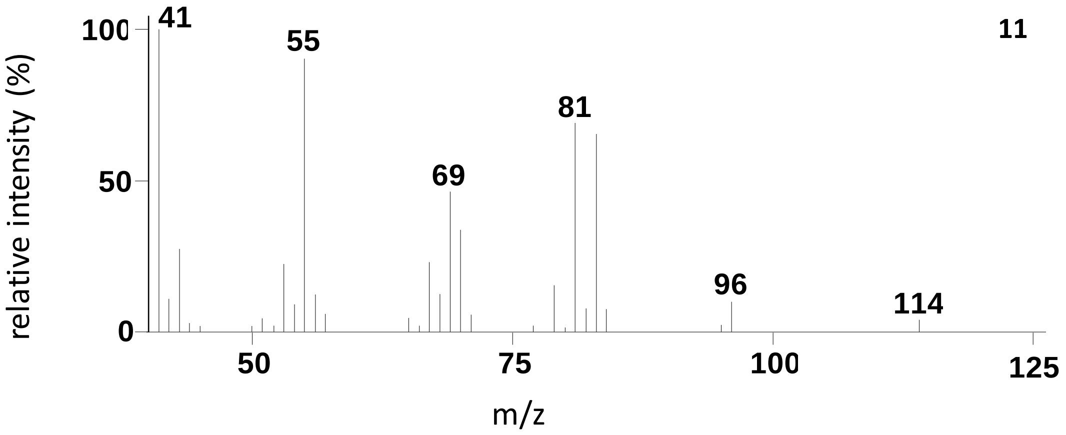

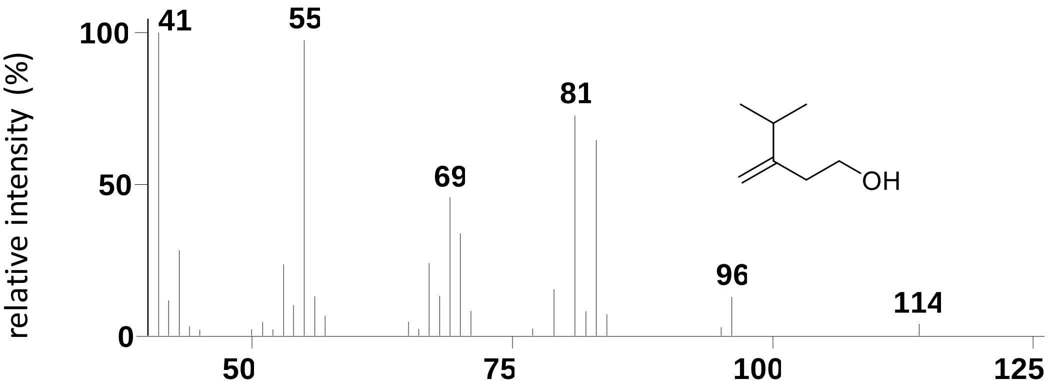

S4K. Comparison of MS of **11** detected (upper) and 5,5-dimethyl-isoprenol (lower).

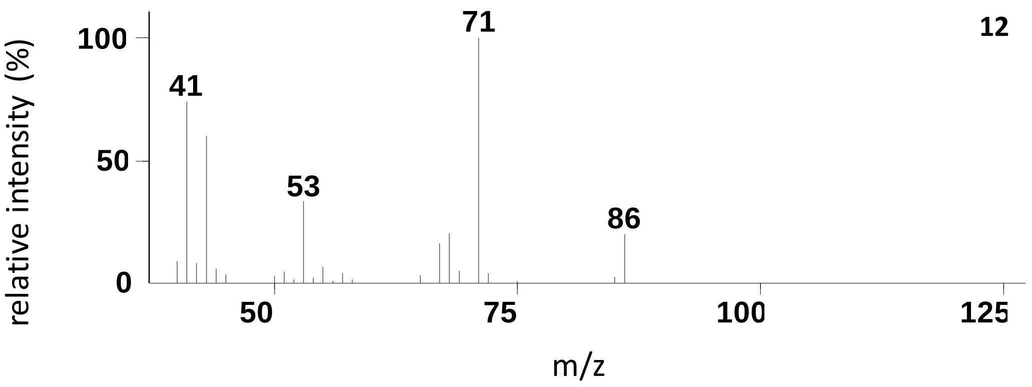

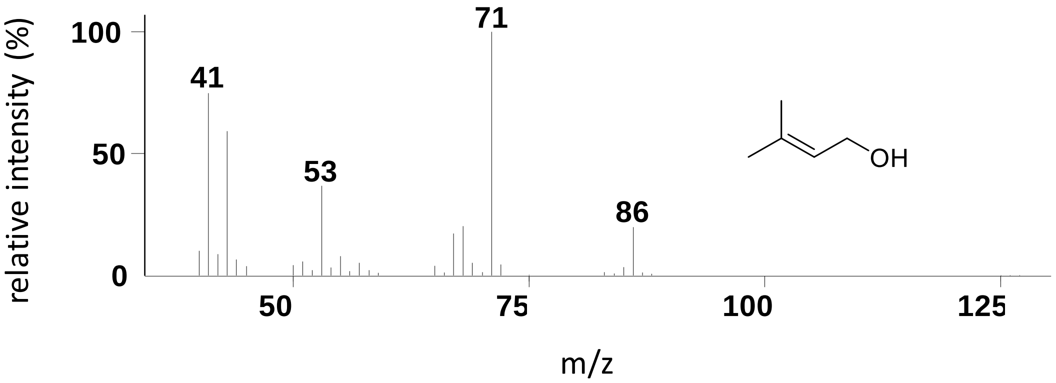

S4L. Comparison of MS of **12** detected (upper) and prenol (lower).

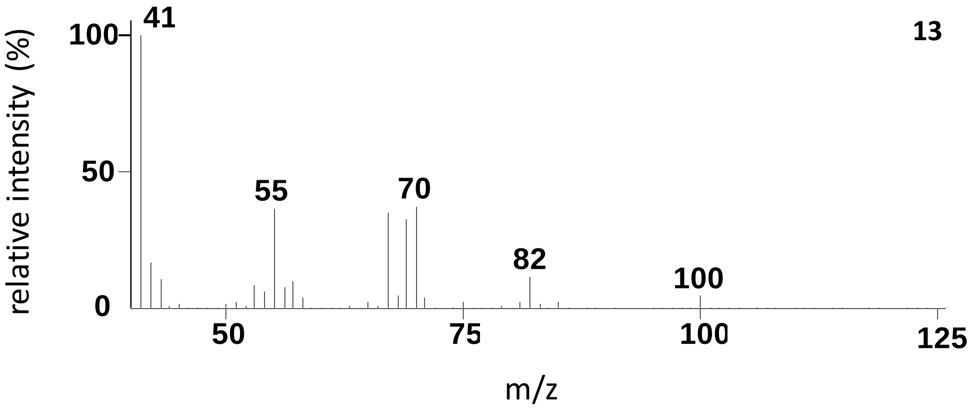

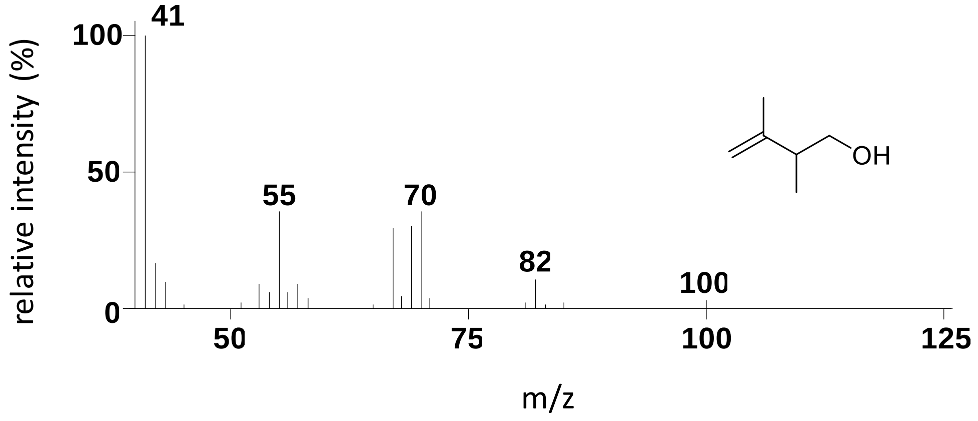

S4M. Comparison of MS of **13** detected (upper) and a mixture of enantiomers of 2-methyl-isoprenol (lower).

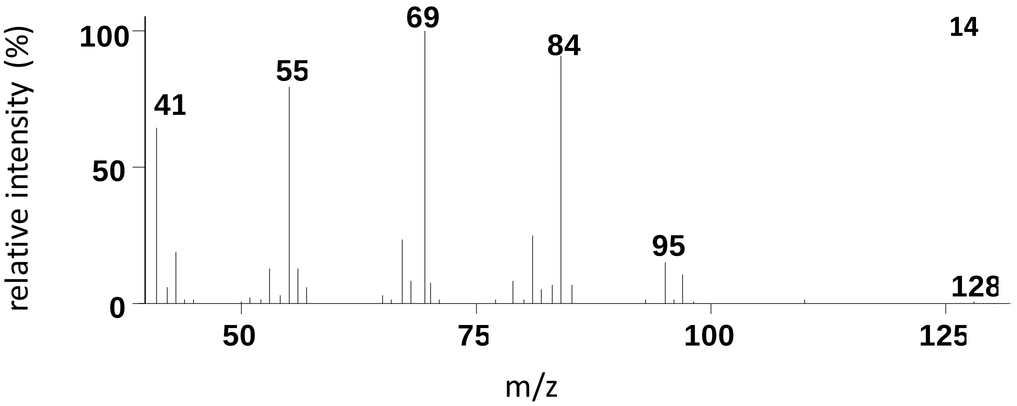

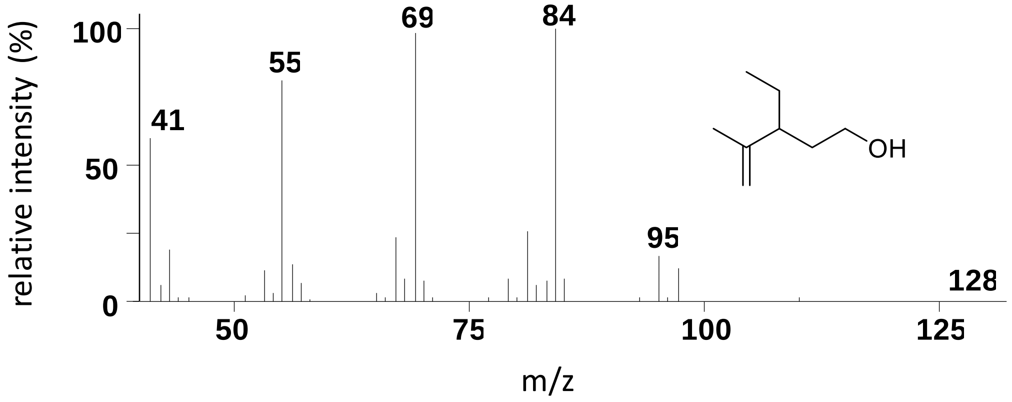

S4N. Comparison of MS of **14** detected (upper) and 3-ethyl-4-methylpent-4-en-1-ol (lower).

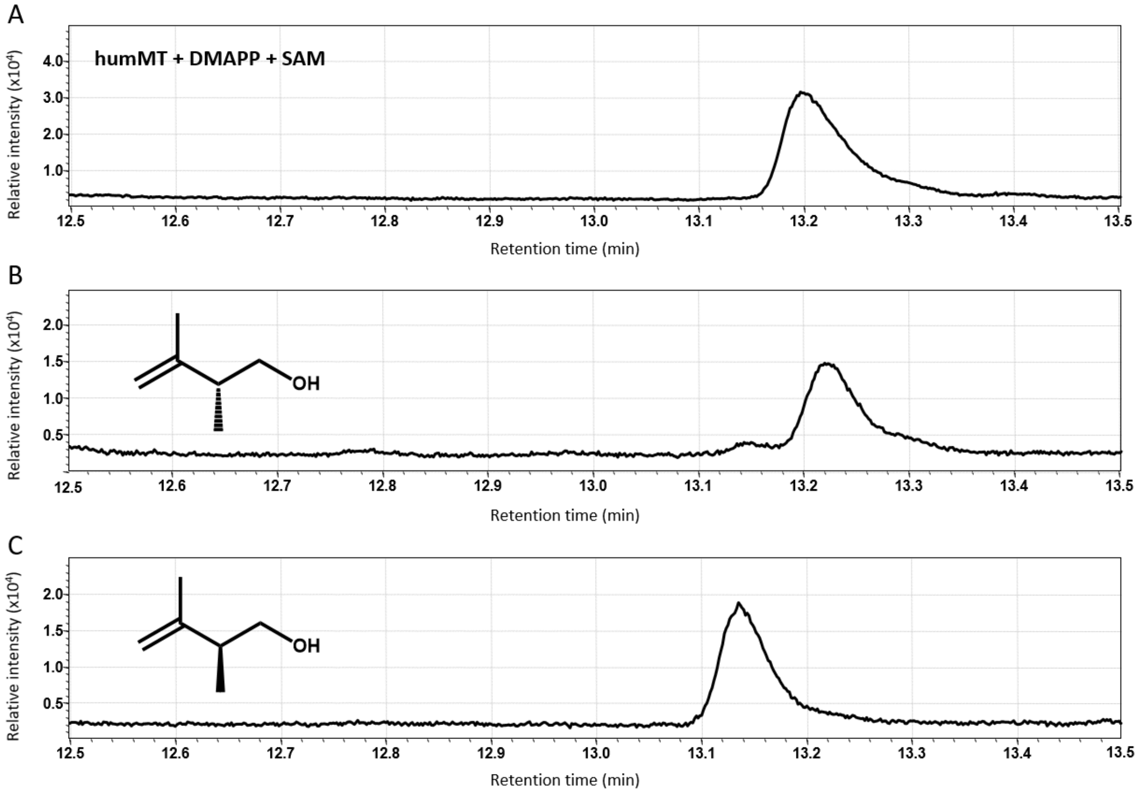

Supplementary Figure S5. Total ion chromatograms of (A) the reaction product from DMAPP incubated with enzyme humMT and SAM followed by incubation with acid phosphatase, (B) incubation of synthetic prenyl pyrophosphate (2*R*)-2-methyl-IPP with acid phosphatase and (C) incubation of synthetic prenyl pyrophosphate (2*S*)-2-methyl-IPP with acid phosphatase.

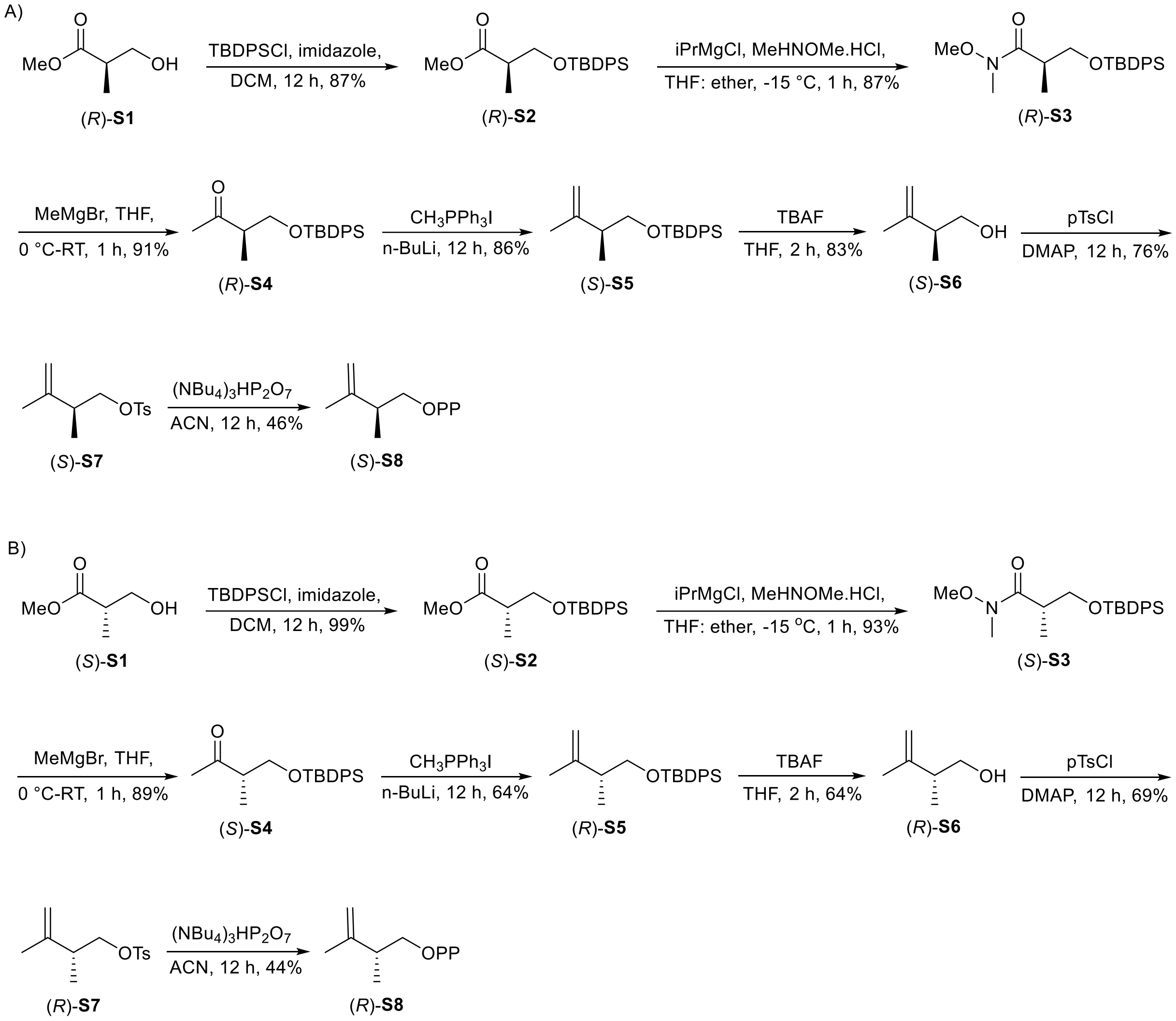

Supplementary Figure S6. Synthesis of A) (2*S*)-2-methyl-IPP and B) (2*R*)-2-methyl-IPP.

S7: Synthesis of (*R*)- and (*S*)-methyl 3-(*tert*-butyldiphenylsilyloxy)-2-methylpropanoate (S2)

In a literature-known procedure[2], the (*R*)-Roche ester ((*R*)-**S1**, 2.00 g, 16.9 mmol, 1.0 eq.) and imidazole (1.62 g, 23.8 mmol, 1.4 eq.) were dissolved in CH_2Cl_2 (100 mL) and the mixture was stirred at room temperature. TBDPSCl (5.67 g, 20.4 mmol, 1.2 eq.) was added dropwise and the reaction mixture was stirred overnight at room temperature. The reaction was quenched by adding H_2O (200 mL). The mixture was extracted three times with diethyl ether (3 x 200 mL). The combined organic layers were dried with $MgSO_4$ and the solvent was removed under reduced pressure. Column chromatography on silica gel [cyclohexane/ethyl acetate (20:1)] resulted in the silylated (*R*)-Roche ester (*R*)-**S2** (5.25 g, 14.7 mmol, 87%) as colorless oil. The same procedure was used to convert (*S*)-Roche ester ((*S*)-**S1**, 2.00 g, 16.9 mmol, 1.0 eq) into (*S*)-**S2** (6.05 g, 16.9 mmol, 99%).

***(R)*-S2:** ^{1}H-NMR (500 MHz, C_6D_6): δ[ppm] = 7.80-7.74 (m, 4H, 4 x CH), 7.26-7.19 (m, 6H, 6 x CH), 3.86 (dd, *J* = 9.7, 7.0 Hz, 1H, 0.5 x CH_2), 3.72 (dd, *J* = 9.7, 5.5 Hz, 1H, 0.5 x CH_2), 3.39

(s, 3H, CH_3), 2.71-2.54 (m, 1H, CH), 1.15 (s, 9H, 3 x CH_3), 1.02 (d, J = 7.1 Hz, 3H, CH_3). ^{13}C-NMR (126 MHz, C_6D_6) δ [ppm] = 174.7 (C_q), 136.0 (CH), 136.0 (CH), 134.0 (C_q), 133.9 (C_q), 130.1(CH), 128.1(CH), 128.1(CH), 66.4(CH_2), 51.2 (CH_3), 42.7(CH), 27.0 (3 x CH_3), 19.5 (C_q), 13.6 (CH_3). TLC [cyclohexane/ethyl acetate (20:1)]: R_f = 0.36. GC (HP-5MS): I = 2243. MS (EI, 70 eV): m/z (%) = 325 (3), 299 (82), 269 (4), 237(9), 213 (100), 199 (16), 197 (16), 183 (56), 181 (26), 153 (20), 135 (13), 105 (24), 91 (10), 77 (8), 57 (9), 41 (9). $[\alpha]_D^{20}$ = –17.2° (c 1.8, CH_2Cl_2).

(*S*)-S2: $[\alpha]_D^{20}$ = +14.6° (c 1.5, CH_2Cl_2). Spectroscopic data as for the (*R*) enantiomer.

S8: Synthesis of (*R*)- and (*S*)-3-(*tert*-butyldiphenylsilyloxy)-*N*-methoxy-*N*,2-dimethylpropanamide (S3)

Ester (*R*)-**S2** (5.25 g, 14.7 mmol, 1.0 eq.) and *N*,*O*-dimethylhydroxylamine hydrochloride (4.28 g, 44.1 mmol, 3.0 eq.) were suspended in THF (65 mL) and cooled to –15 °C. A solution of *i*PrMgCl (2 M in Et_2O, 18.4 mL, 36.8 mmol, 2.5 eq.) was added dropwise and the reaction mixture was stirred at –15 °C for 1 h. After completion of the reaction (monitored by TLC), the reaction was quenched by addition of sat. aq. NH_4Cl (25 mL) at 0 °C. The mixture was extracted three times with EtOAc (3 x 60 mL). The combined organic layers were washed with sat. NaCl (40 mL), dried over $MgSO_4$, and concentrated in vacuo. Column chromatography on silica gel [cyclohexane/ethyl acetate (5:1)] gave the amide (*R*)-**S3** (4.92 g, 12.8 mmol, 87%) as white solid. The same procedure was used to convert ester (*S*)-**S2** (6.05 g, 16.9 mmol, 1.0 eq.) into (*S*)-**S3** (6.05 g, 15.7 mmol, 93%).

(*R*)-S3: ^{1}H-NMR (500 MHz, C_6D_6): δ [ppm] = 7.89-7.85 (m, 2H, 2 x CH), 7.83-7.79 (m, 2H, 2 x CH), 7.29-7.19 (m, 6H, 6 x CH), 4.20 (dd, J = 9.5, 8.5 Hz, 1H, 0.5 x CH_2), 3.68 (dd, J = 9.4, 5.8 Hz, 1H, 0.5 x CH_2), 3.24 (m, 1H, CH), 3.16 (s, 3H, CH_3), 2.93 (s, 3H, CH_3), 1.18 (s, 9H, 3 x CH_3), 1.04 (d, J = 6.9 Hz, 3H, CH_3). ^{13}C-NMR (126 MHz, C_6D_6) δ [ppm] = 175.9 (C_q), 136.2 (2 x CH), 136.0 (2 x CH), 134.4 (C_q), 133.9 (C_q), 130.0 (CH), 130.0 (CH), 128.1 (2 x CH), 128.1 (2 x CH), 66.9 (CH_2), 61.1 (CH), 38.4 (CH_3), 32.1 (CH), 27.1 (3 x CH_3), 19.5 (C_q), 14.0 (CH_3). TLC [cyclohexane/ethyl acetate (5:1)]: R_f = 0.35. GC (HP-5MS): I = 2497. MS (EI, 70 eV): m/z (%) = 328 (100), 296 (6), 213(6), 199 (26), 197 (16), 183 (12), 181 (11), 135 (14), 105 (9), 91 (4), 82 (4), 77 (6), 57 (7), 41 (6). $[\alpha]_D^{20}$ = –13.9° (c 1.8, CH_2Cl_2).

(*S*)-S3: $[\alpha]_D^{20}$ = +10.2° (c 1.6, CH_2Cl_2). Spectroscopic data as for the (*R*) enantiomer.

S9 Synthesis of (*R*)- and (*S*)-4-(*tert*-butyldiphenylsilyloxy)-3-methylbutan-2-one (S4)

Amide (*R*)-**S3** (4.92 g, 12.8 mmol, 1.0 eq.) was dissolved in THF (50 mL) and the mixture was stirred at 0 °C until it dissolved. The MeMgBr (3 M in Et_2O,12.8 mL, 38.4 mmol, 3.0 eq.) was added dropwise and the reaction mixture was stirred at 0 °C for 1 h. After completion of the reaction (monitored by TLC), the reaction was quenched by addition of sat. aq. NH_4Cl (25 mL) at 0 °C. The mixture was extracted three times with EtOAc (3 x 80 mL). The combined organic layers were washed with sat. NaCl (20 mL), dried over $MgSO_4$, and concentrated in vacuo. Column chromatography on silica gel [cyclohexane/ethyl acetate (20:1)] gave the methyl ketone (*R*)-**S4** (3.94 g, 11.6 mmol, 91%) as colorless oil. The same procedure was used to convert amide (*S*)-**S3** (6.05 g, 15.7 mmol, 1.0 eq.) into (*S*)-**S4** (4.75 g, 14.0 mmol, 89%).

(*R*)-S4: ^{1}H-NMR (500 MHz, C_6D_6): δ [ppm] = 7.78-7.70 (m, 4H, 4 x CH), 7.26-7.19 (m, 6H, 6 x CH), 3.78 (dd, J = 10.0, 7.3 Hz, 1H, 0.5 x CH_2), 3.60 (dd, J = 10.0, 5.3 Hz, 1H, 0.5 x CH_2), 2.52-2.38 (m, 1H, CH), 1.85 (s, 3H, CH_3), 1.13 (s, 9H, 3 x CH_3), 0.82 (d, J = 7.0 Hz, 3H, CH_3). ^{13}C-NMR (126 MHz, C_6D_6) δ [ppm] = 208.8 (C_q), 136.0 (2 x CH), 136.0 (2 x CH), 133.9

(C_q), 133.8 (C_q), 130.1 (2 x CH), 128.1 (4 x CH), 66.4 (CH_2), 49.2 (CH), 29.0 (CH_3), 27.0 (3 x CH_3), 19.5 (C_q), 12.9 (CH_3). TLC [cyclohexane/ethyl acetate (20:1)]: R_f = 0.26. GC (HP-5MS): *I* = 2239. MS (EI, 70 eV): *m*/*z* (%) = 283 (96), 253 (13), 239 (50), 205 (61), 199 (74), 197 (25), 187 (23), 183 (100), 181 (43), 175 (20), 135 (16), 123 (25), 105 (25), 77 (22), 43 (28). $[\alpha]_D^{20}$ = –18.0° (*c* 1.5, CH_2Cl_2).

(*S*)-S4: $[\alpha]_D^{20}$ = +20.9° (*c* 1.8, CH_2Cl_2). Spectroscopic data as for the (*R*) enantiomer.

S10: Synthesis of (*S*)- and (*R*)-*tert*-butyl((2,3-dimethylbut-3-en-1-yl)oxy)diphenylsilane (S5)

CH_3PPh_3I (8.44 g, 20.9 mmol, 1.8 eq.) was suspended in THF (100 mL) and cooled to 0 °C. The *n*-BuLi (1.6 M in hexane, 13.0 mL, 20.8 mmol, 1.8 eq.) was added dropwise and the mixture was stirred at 0 °C for 1 h. After cooling to the mixture to –78 °C, the ketone (*R*)-**S4** (3.94 g, 11.6 mmol, 1.0 eq.) was added dropwise and the reaction mixture was stirred at room temperature overnight. The reaction was quenched by pouring ice-water mixture (200 mL). The mixture was extracted three times with diethyl ether (3 x 200 mL). The combined organic layers were washed with sat. NaCl (20 mL), dried over $MgSO_4$, and concentrated under reduced pressure. Column chromatography on silica gel [cyclohexane/ethyl acetate (100:1)] gave the olefin (*S*)-**S5** (3.38 g, 10.0 mmol, 86%) as colorless oil. The same procedure was used to convert ketone (*S*)-**S4** (4.75 g, 14.0 mmol, 1.0 eq.) into (*R*)-**S5** (3.03 g, 8.95 mmol, 64%).

(*S*)-S5: ^{1}H-NMR (500 MHz, C_6D_6): δ[ppm] = 7.84-7.73 (m, 4H, 4 x CH), 7.27-7.20 (m, 6H, 6 x CH), 4.83-4.79 (m, 2H, CH_2), 3.70 (dd, *J* = 9.9, 6.3 Hz, 1H, 0.5 x CH_2), 3.57 (dd, *J* = 9.9, 6.8 Hz, 1H, 0.5 x CH_2), 2.44-2.32 (m, 1H, CH), 1.57 (t, *J* = 1.2 Hz, 3H, CH_3), 1.19 (s, 9H, 3 x CH_3), 1.03 (d, *J* = 7.0 Hz, 3H, CH_3). ^{13}C-NMR (126 MHz, C_6D_6) δ[ppm] = 147.7 (C_q), 136.1 (2 x CH), 136.1 (2 x CH), 134.4 (C_q), 134.4 (C_q), 130.0 (2 x CH), 128.1 (4 x CH), 111.0 (CH_2), 68.0 (CH_2), 43.8 (CH), 27.2 (3 x CH_3), 20.6 (CH_3), 19.6 (C_q), 16.3 (CH_3). TLC [cyclohexane/ethyl acetate (100:1)]: R_f = 0.40. GC (HP-5MS): *I* = 2122. MS (EI, 70 eV): *m*/*z* (%) = 281 (37), 239 (100), 211 (8), 203 (10), 199 (15), 197 (13), 135 (16), 121 (8), 105 (14), 77 (8), 57 (10), 41 (15). $[\alpha]_D^{20}$ = –0.1° (*c* 1.8, CH_2Cl_2).

(*R*)-S5: $[\alpha]_D^{20}$ = +0.2° (*c* 1.3, CH_2Cl_2). Spectroscopic data as for the (*S*) enantiomer.

S11: Synthesis of (*S*)- and (*R*)-2,3-dimethylbut-3-en-1-ol (S6)

Olefin (*S*)-**S5** (3.38 g, 10.0 mmol, 1.0 eq.) was suspended in THF (45 mL) and the solution was cooled to 0 °C. TBAF (1 M in THF, 12.0 mL, 12.0 mmol, 1.2 eq.) was added dropwise and the reaction mixture was stirred at room temperature for 2 h. The reaction was quenched by addition of water (50 mL). The mixture was extracted three times with diethyl ether (3 x 100 mL). The combined organic layers were washed with sat. NaCl (10 mL), dried over $MgSO_4$, and concentrated under reduced pressure (700 mbar, 40 °C, 20 min). Column chromatography on silica gel [*n*-pentane/diethyl ether (1:1)] gave the alcohol (*S*)-**S6** (0.83 g, 8.3 mmol, 83%) as colorless oil. The same procedure was used to convert olefin (*R*)-**S5** (3.03 g, 8.95 mmol, 1.0 eq.) into (*R*)-**S6** (0.57 g, 5.69 mmol, 64%).

(*S*)-S6: ^{1}H-NMR (500 MHz, C_6D_6): δ [ppm] = 4.79-4.74 (m, 1H, CH), 4.73- 4.70 (m, 1H, CH), 3.35-3.29 (m, 1H, 0.5 x CH_2), 3.29-3.24 (m, 1H, 0.5 x CH_2), 2.19-2.08 (m, 1H, CH), 1.51 (t, *J* =

1.2 Hz, 3H, CH_3), 0.87 (d, *J* = 6.9 Hz, 3H, CH_3). ^{13}C-NMR (126 MHz, C_6D_6) δ [ppm] = 147.3 (C_q), 111.4 (CH_2), 65.7 (CH_2), 43.9 (CH), 19.9 (CH_3), 15.8 (CH_3). TLC [*n*-pentane/diethyl ether (1:1)]: R_f = 0.50. GC (HP-5MS): *I* < 800. MS (EI, 70 eV): *m*/*z* (%) = 100 (5), 82 (11), 70 (37), 69 (37), 67 (36), 55 (43), 53 (18), 41 (100), 39 (41). $[\alpha]_D^{20}$ = –9.0° (*c* 0.67, CH_2Cl_2).

(*R*)-S6: $[\alpha]_D^{20}$ = +10.5° (c = 0.6, CH_2Cl_2). Lit: $[\alpha]_D^{22}$ = +9.0° (*c* 3.0, $CHCl_3$).[3] Spectroscopic data as for the (*S*) enantiomer.

S12: Synthesis of (*S*)- and (*R*)-2,3-dimethylbut-3-en-1-yl 4-methylbenzenesulfonate (S7)

Alcohol (*S*)-**S6** (0.83 g, 8.3 mmol, 1.0 eq.) was suspended in CH_2Cl_2 (80 mL) and the solution was cooled to 0 °C. After adding of DMAP (3.35 g, 27.4 mmol, 3.3 eq.), *p*-TsCl (partially dissolved in 10 mL CH_2Cl_2, 3.96 g, 20.8 mmol, 2.5 eq.) was added dropwise to the solution and the reaction mixture was stirred at room temperature over night. The reaction was quenched by addition of sat. NH_4Cl (100 mL). The mixture was extracted three times with diethyl ether (3 x 100 mL). The combined organic layers were washed with sat. NaCl (10 mL), dried over $MgSO_4$, and concentrated under reduced pressure. Column chromatography on silica gel [cyclohexane/ethyl acetate (10:1)] gave the tosylate (*S*)-**S7** (1.60 g, 6.29 mmol, 76%) as colorless oil. The same procedure was used to convert alcohol (*R*)-**S6** (0.57 g, 5.69 mmol, 1.0 eq.) into (*R*)-**S7** (1.00 g, 3.93 mmol, 69%).

(*S*)-S7: ^{1}H-NMR (500 MHz, C_6D_6): δ [ppm] = 7.76 (d, *J* = 8.3 Hz, 2H, 2 x CH), 6.69 (d, *J* = 8.0, 0.7 Hz, 2H, 2 x CH), 4.69-4.64 (m, 1H, 0.5 x CH_2), 4.62-4.57 (m, 1H, 0.5 x CH_2), 3.91 (dd, *J* = 9.6, 6.7 Hz, 1H, CH), 3.79 (dd, *J* = 9.6, 6.9 Hz, 1H, CH), 2.29-2.17 (m, 1H, CH), 1.82 (s, 3H, CH_3), 1.37 (dd, *J* = 1.5, 0.8 Hz, 3H, CH_3), 0.76 (d, *J* = 7.0 Hz, 3H, CH_3). ^{13}C-NMR (126 MHz, C_6D_6) δ [ppm] = 145.3 (C_q), 144.2(C_q), 134.5(C_q), 129.8(2 x CH), 128.2(2 x CH), 112.1(CH_2), 72.9 (CH_2), 40.4 (CH), 21.1 (CH_3), 20.0 (CH_3), 15.8 (CH_3). TLC [cyclohexane/ethyl acetate (10:1)]: R_f = 0.38. GC (HP-5MS): *I* = 1879. MS (EI, 70 eV): *m*/*z* (%) = 173 (6), 155 (58), 91 (100), 82 (90), 69 (28), 67 (39), 65 (40), 41 (41), 39 (23). $[\alpha]_D^{20}$ = +7.0° (c = 1.0, CH_2Cl_2). Lit: $[\alpha]_D^{27}$ = +7.4° (*c* 0.91, $CHCl_3$).[4]

(*R*)-S7: $[\alpha]_D^{20}$ = –6.5° (*c* 0.8, CH_2Cl_2). Lit: $[\alpha]_D^{25}$ = –6.1° (*c* 5.5, $CHCl_3$).[5] Spectroscopic data as for the (*S*) enantiomer.

S13: Synthesis of (*S*)- and (*R*)-2,3-dimethylbut-3-en-1-yl diphosphate (S8)

Following a known pyrophosphorylation method[6], $(^nNBu_4)_3HP_2O_7$ (8.52 g, 9.44 mmol, 1.5 eq.) was added to acetonitrile (2 mL), followed by the dropwise addition of the tosylate (*S*)-**S7** (1.60 g, 6.29 mmol,1.0 eq., in 6 mL acetonitrile). The reaction mixture was stirred overnight, and the solvent was removed under reduced pressure. The residue was loaded onto an ion exchange resin column (DOWEX® 50W-X8, 100-200 mesh, NH_4^+ form), followed by elution with two column volumes of elution buffer (25 mM NH_4HCO_3 in 2% ^{i}PrOH/H_2O). The eluate was lyophilized, the residue was dissolved in NH_4HCO_3 solution (0.1 M, 8 mL) and mixed with 1:1 MeCN/^{i}PrOH (20 mL). The precipitate was separated by centrifugation (2000 x g, 5 min) and the liquid phase containing the target compound was collected. The procedure of dissolving the precipitate in NH_4HCO_3 solution (0.1 M, 8 mL) and mixing with 1:1 MeCN/^{i}PrOH (20 mL) followed by centrifugation was repeated three times, before the pooled liquid fractions were concentrated under reduced pressure. The residue was taken up in water (6 mL) and lyophilized again to yield the diphosphate (*S*)-**S8** as an inseparable 1:1.7 mixture (by NMR peak integration) with ammonium tosylate as a white solid (0.90 g, 2.89 mmol, 46%). The same procedure was used to convert tosylate (*R*)-**S7** (1.00 g, 3.93 mmol, 69%) into the (*R*)-**S8** as an

inseparable 1:1.7 mixture (by NMR peak integration) with ammonium tosylate as a white solid (0.54 g, 1.73 mmol, 44%).

(*S*)-S8: ^{1}H-NMR (500 MHz, D_2O): δ [ppm] = 4.86-4.83 (m, 2H, CH_2), 3.93-3.87 (m, 1H, 0.5 x CH_2), 3.87-3.80 (m, 1H, 0.5 x CH_2), 2.59-2.45 (m, 1H, CH), 1.74 (s, 3H, CH_3), 1.04 (dd, J = 6.9, 1.4 Hz, 3H, CH_3). ^{13}C-NMR (126 MHz, D_2O) δ [ppm] = 148.9 (C_q), 110.6 (CH_2), 68.9 (d, $^{2}J_{C,P}$ = 6.0 Hz, CH_2), 41.2 (d, $^{3}J_{C,P}$ = 7.6 Hz, CH), 19.2 (CH_3), 15.5 (CH_3). ^{13}P-NMR (202 MHz, D_2O): δ [ppm] = –7.32 (d, $^{2}J_{P,P}$ = 21.3 Hz, 1P), –10.47 (d, $^{2}J_{P,P}$ = 21.3 Hz, 1P). HRMS (TOF): m/z = 259.0146 (calc. for $[C_6H_{13}O_7P_2]^-$ 259.0142).

(*R*)-S8: HRMS (TOF): m/z = 259.0145 (calc. for $[C_6H_{13}O_7P_2]^-$ 259.0142). Spectroscopic data as for the (*S*) enantiomer.

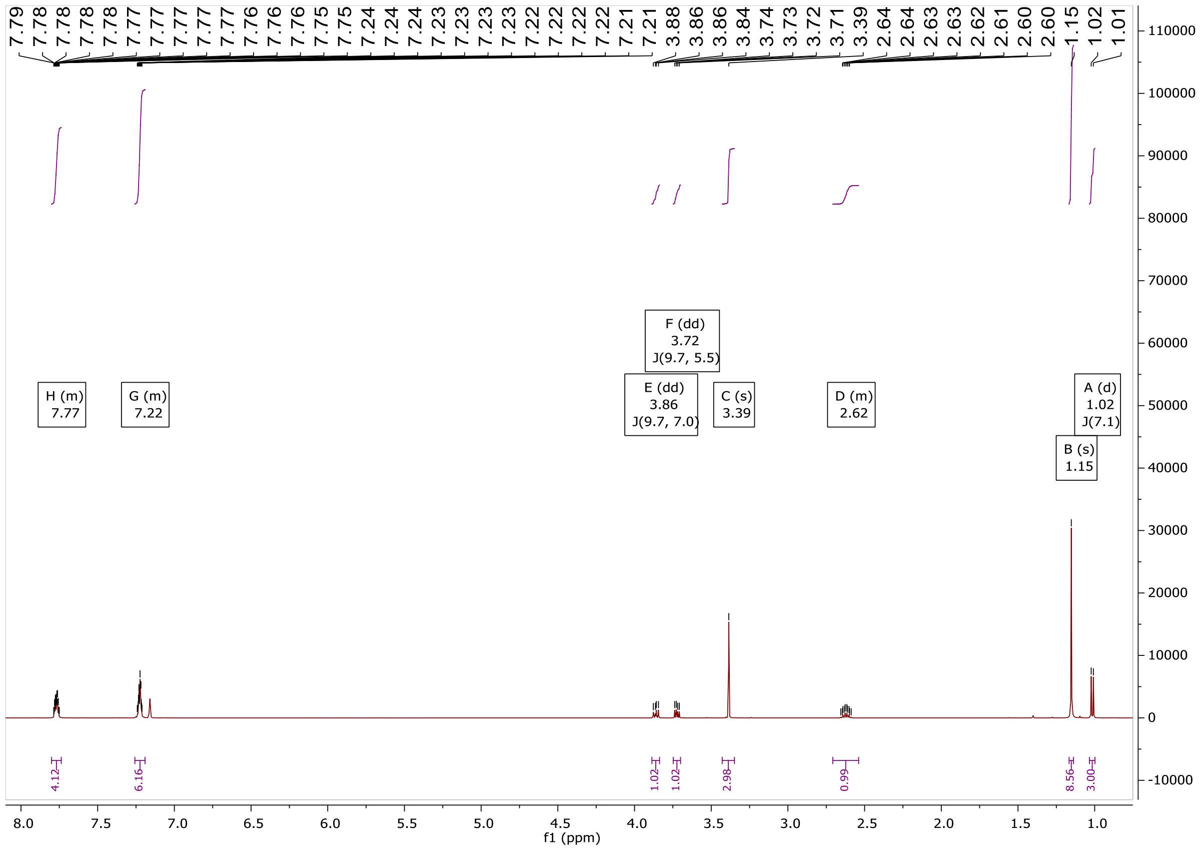

Supplementary Figure S7. ^{1}H-NMR (500 MHz, C_6D_6) of **S2**.

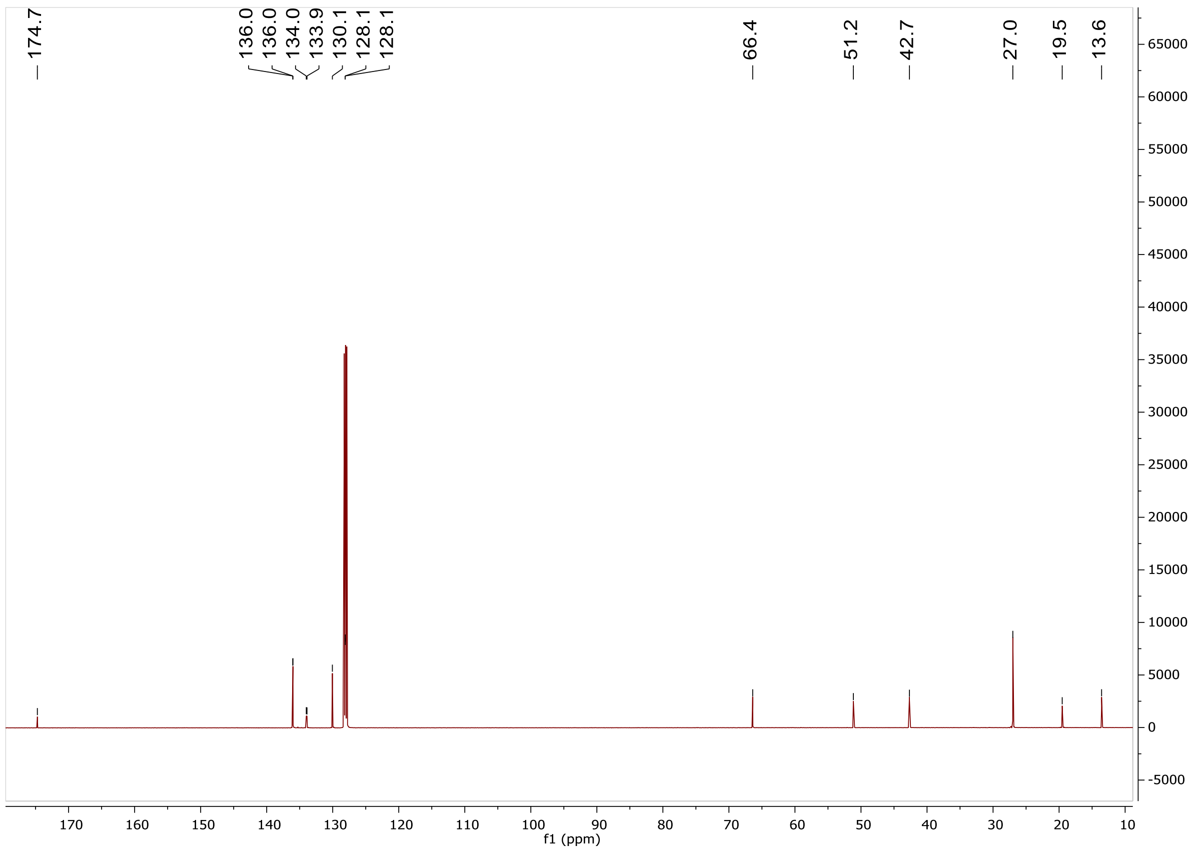

Supplementary Figure S8. ^{13}C-NMR (126 MHz, C_6D_6) of **S2**.

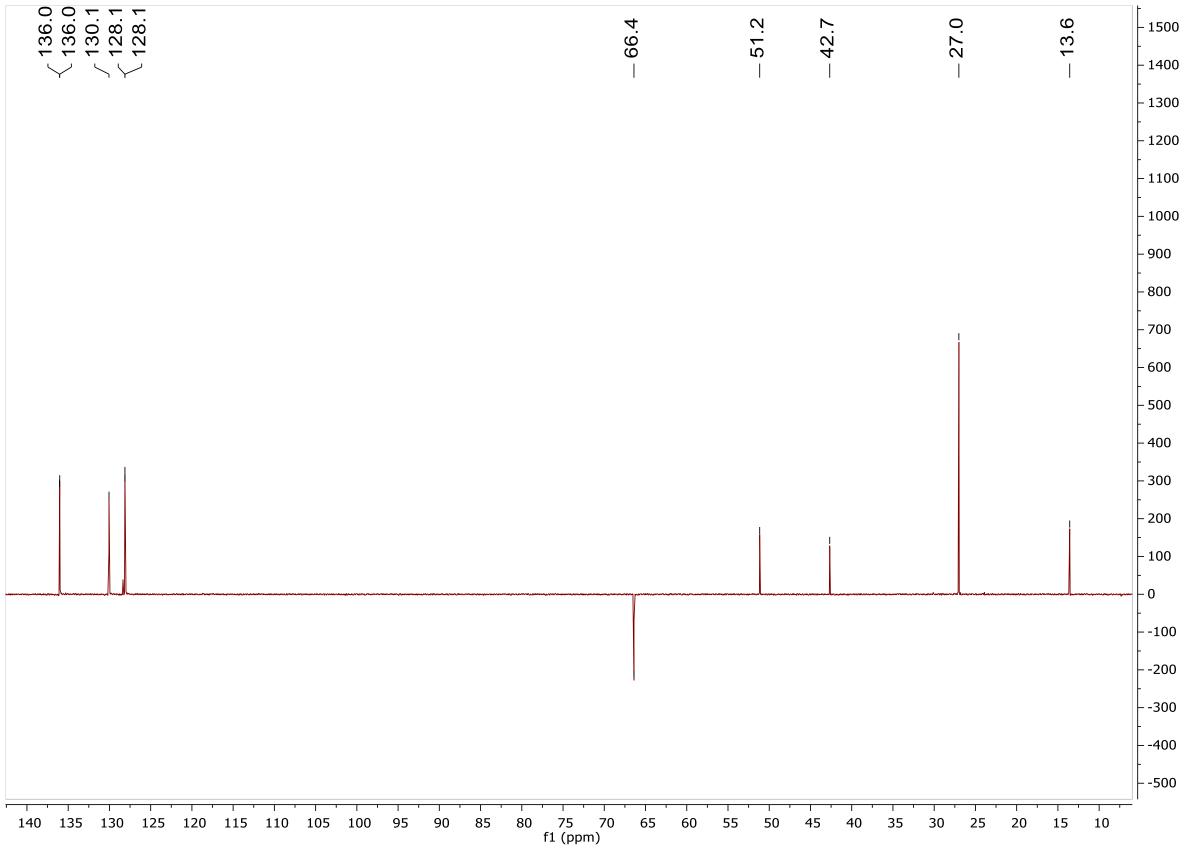

Supplementary Figure S9. DEPT135 (126 MHz, C_6D_6) of **S2**.

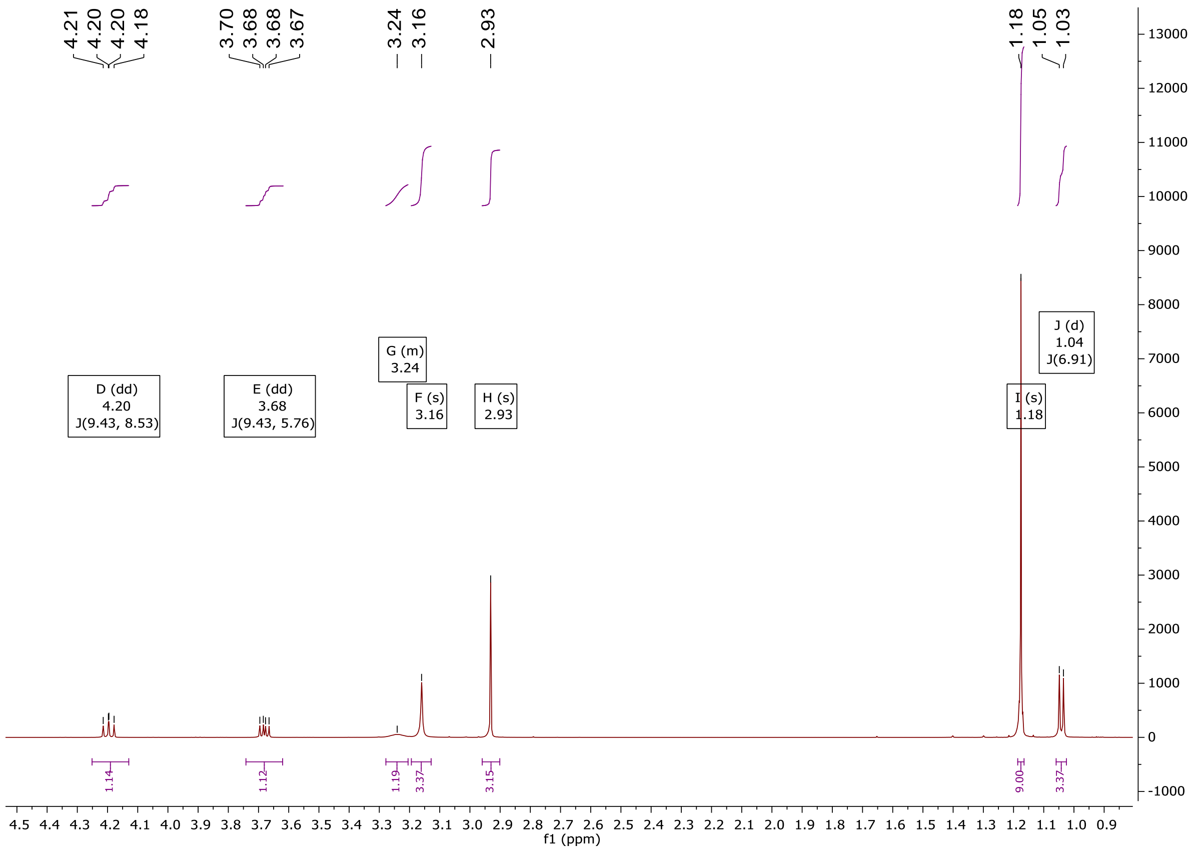

Supplementary Figure S10. ^{1}H-NMR (500 MHz, C_6D_6) of **S3**.

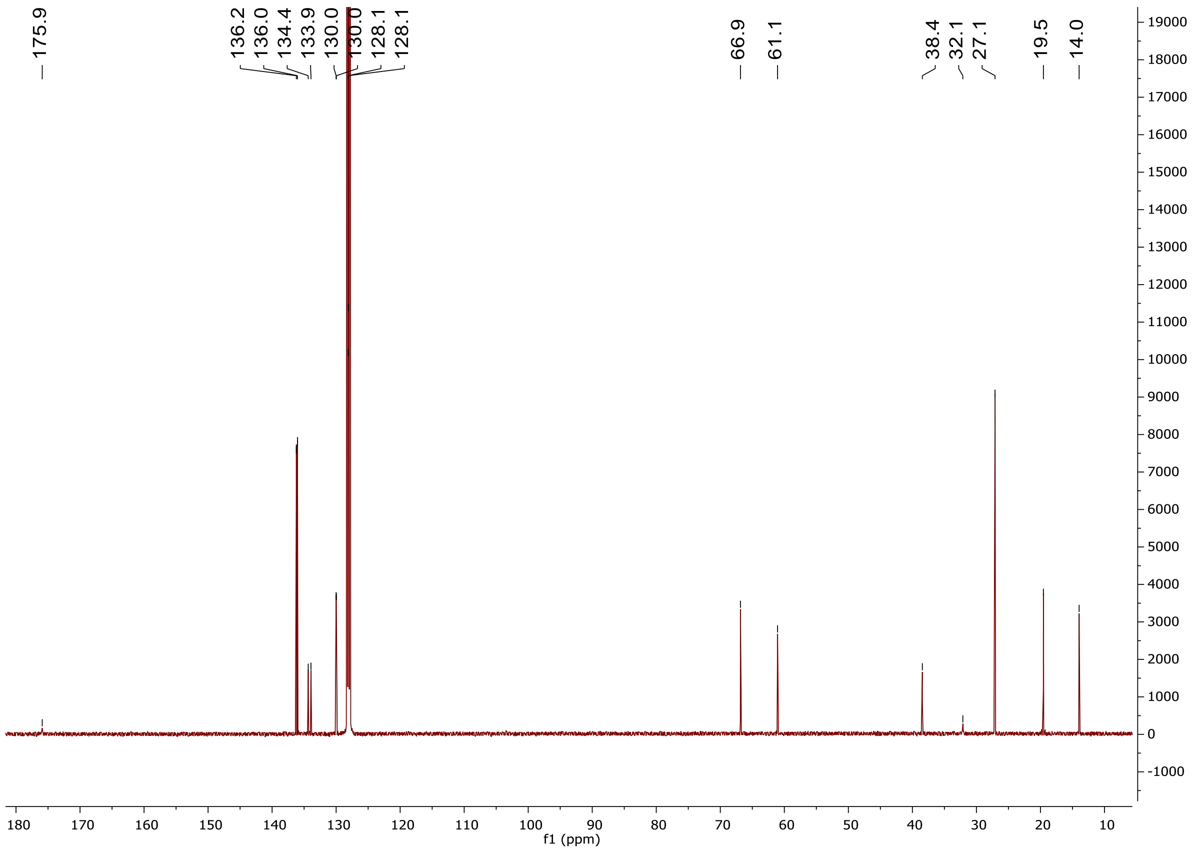

Supplementary Figure S11. ^{13}C-NMR (126 MHz, C_6D_6) of **S3**.

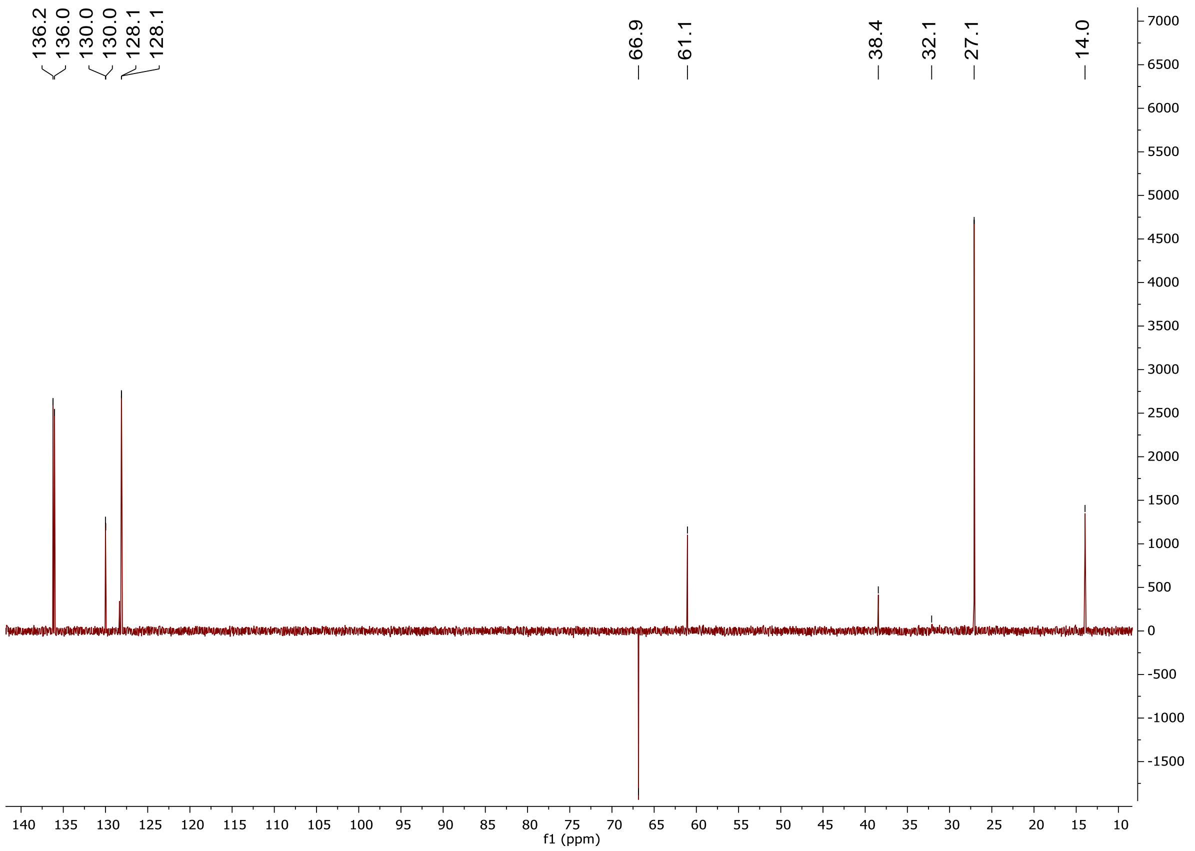

Supplementary Figure S12. DEPT135 (126 MHz, C_6D_6) of **S3**.

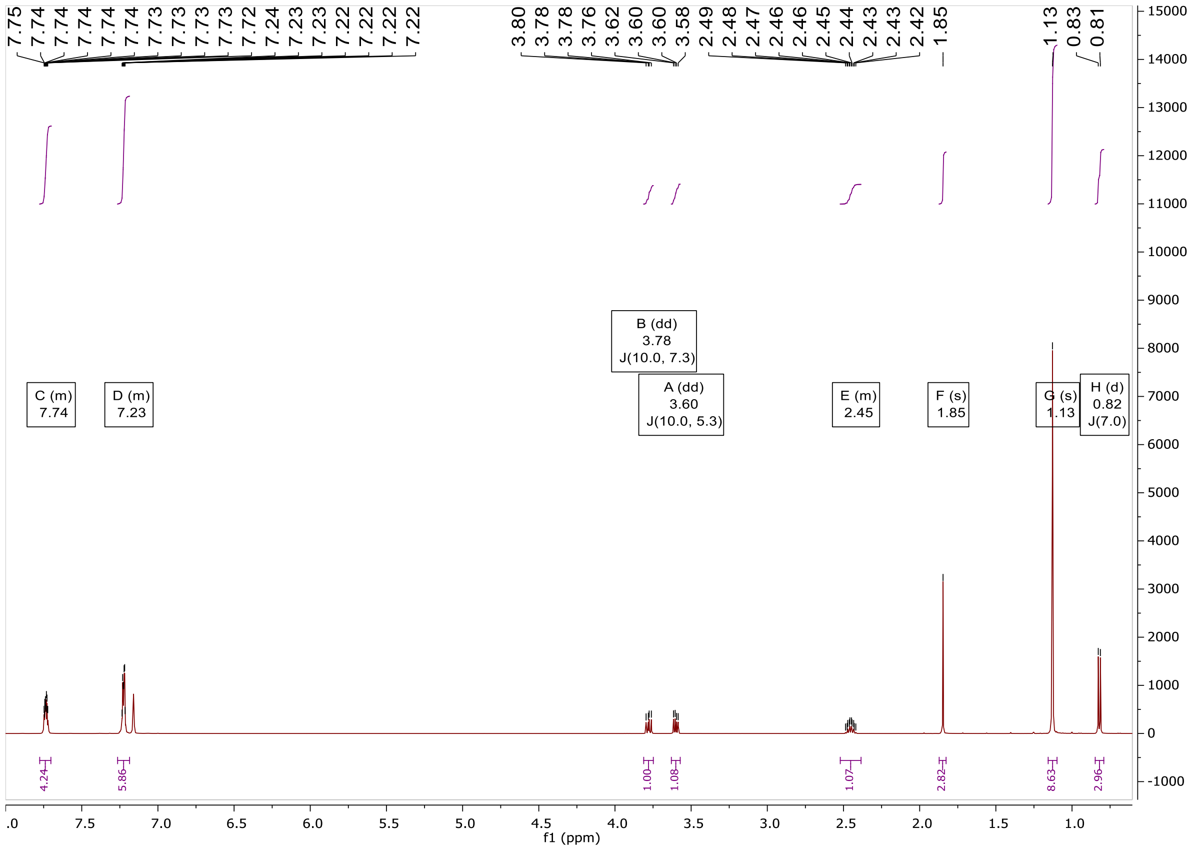

Supplementary Figure S13. ^{1}H-NMR (500 MHz, C_6D_6) of **S4**.

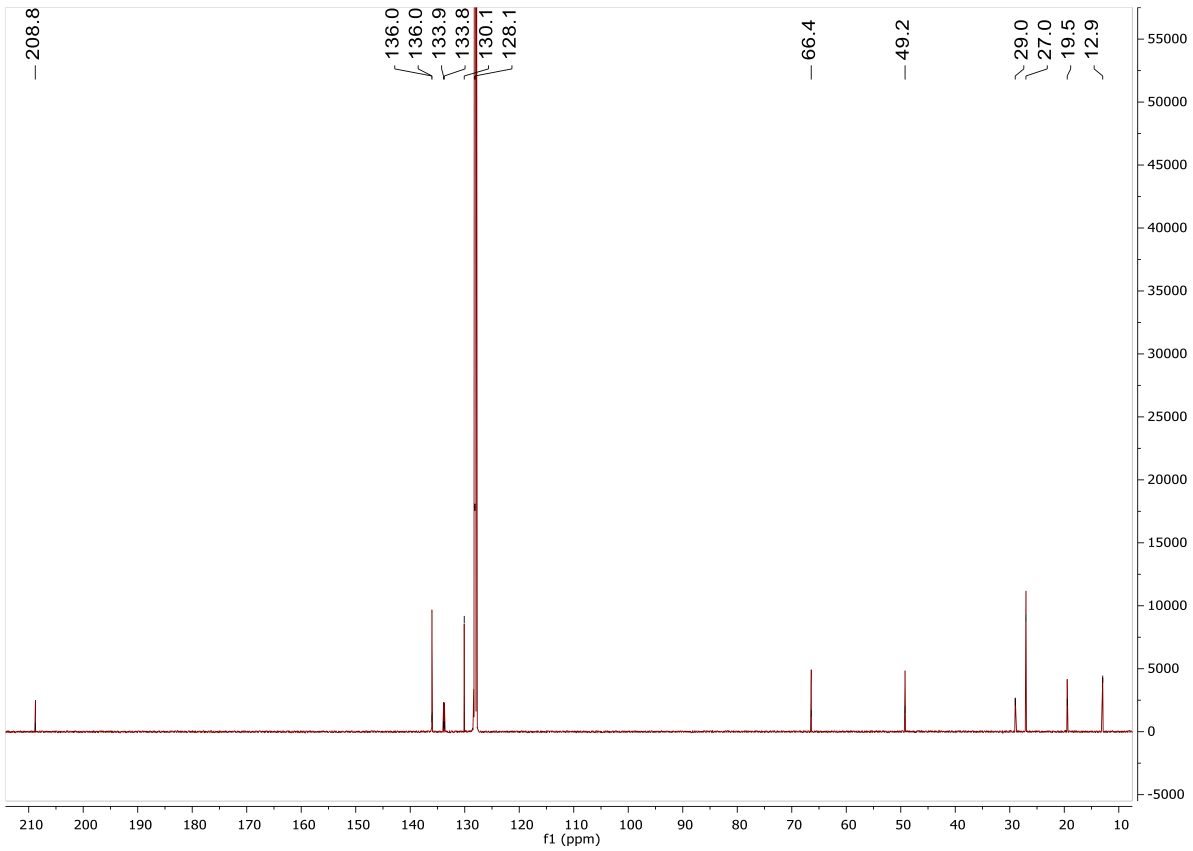

Supplementary Figure S14. ^{13}C-NMR (126 MHz, C_6D_6) of **S4**.

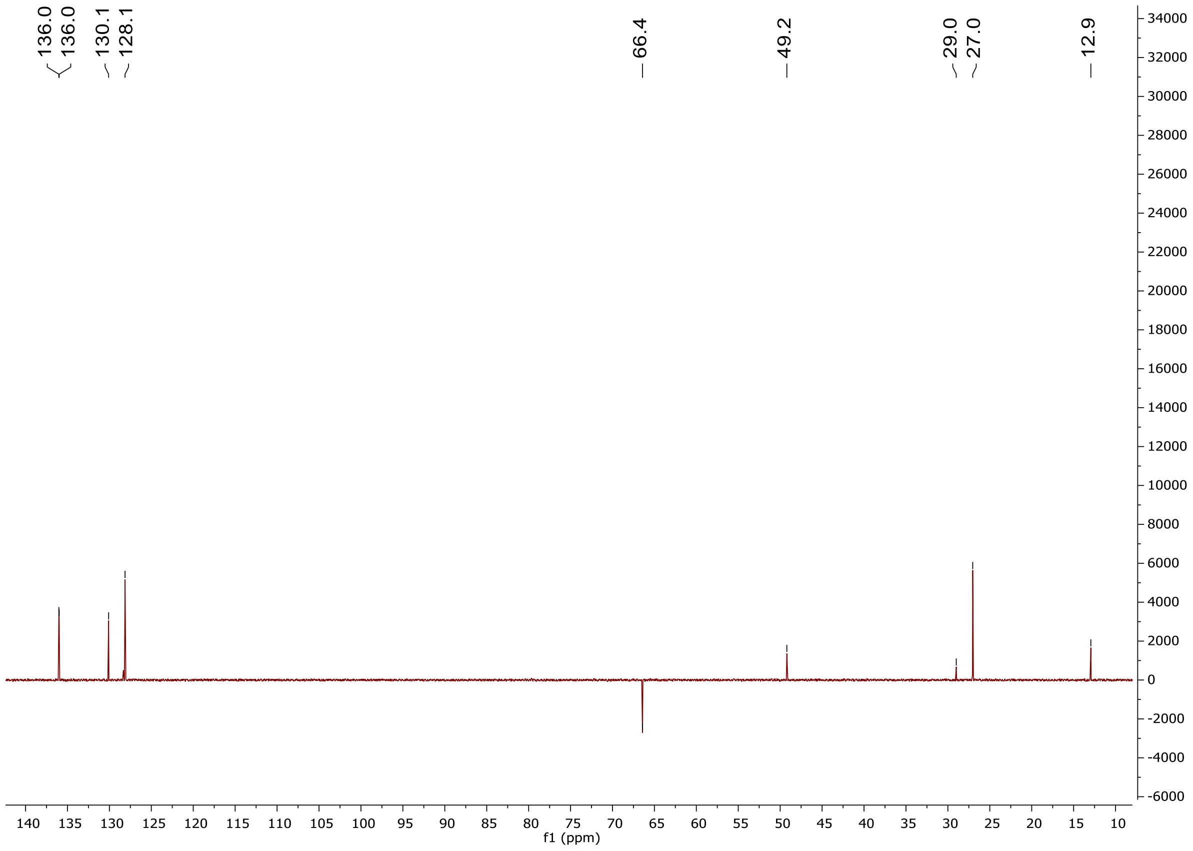

Supplementary Figure S15. DEPT135 (126 MHz, C_6D_6) of **S4**.

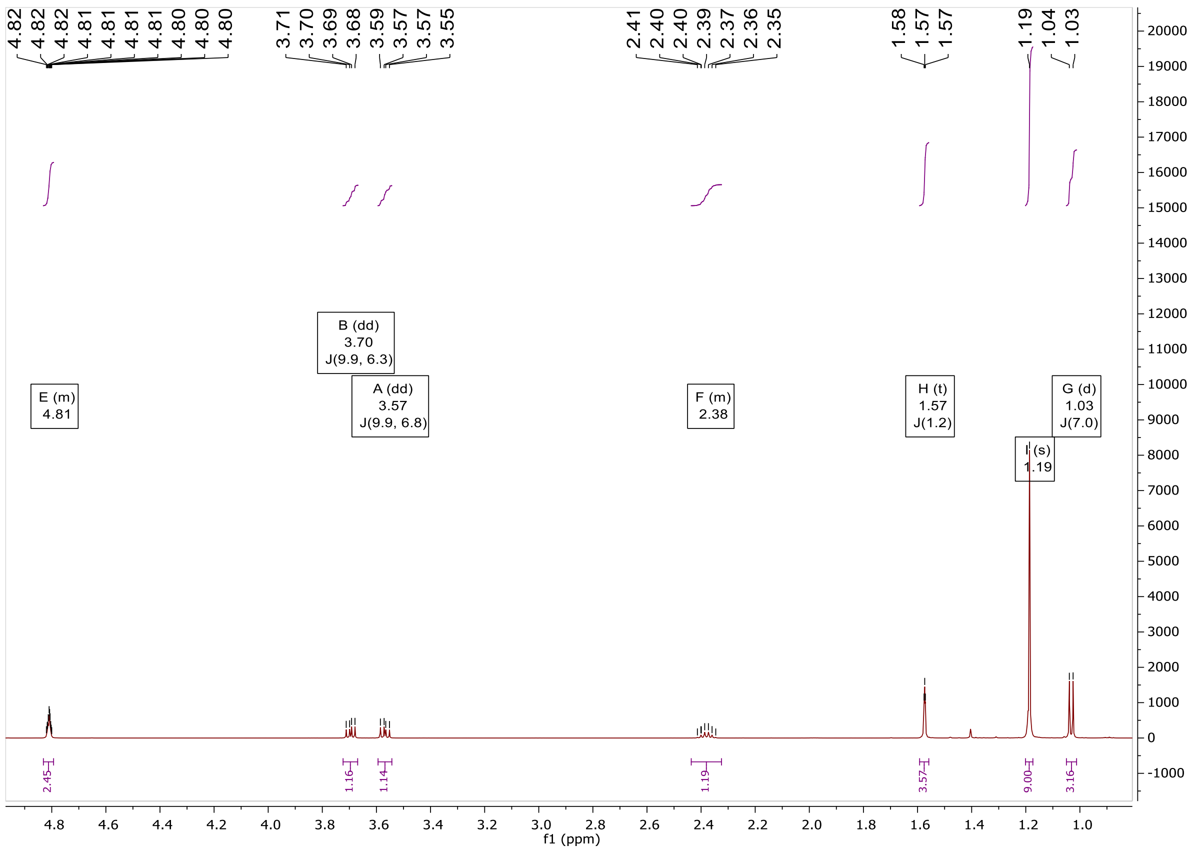

Supplementary Figure S16. ^{1}H-NMR (500 MHz, C_6D_6) of **S5**.

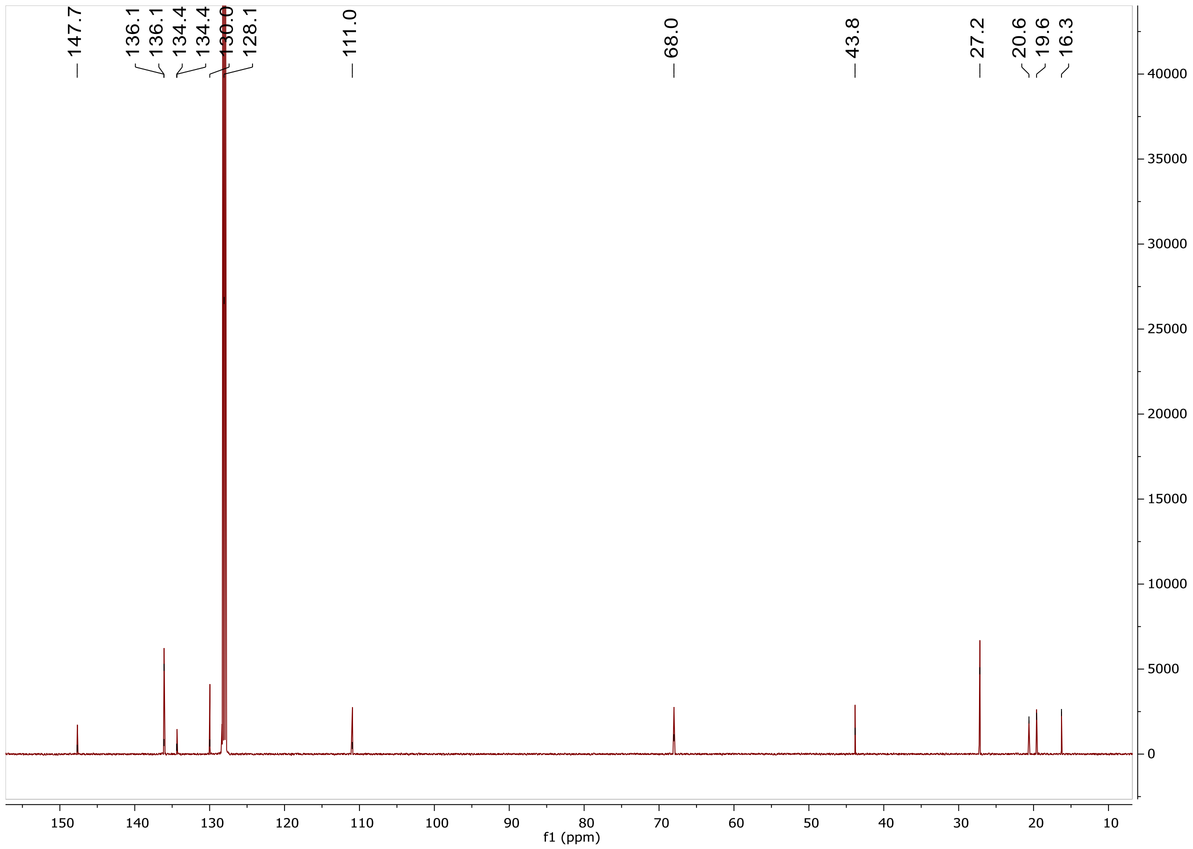

Supplementary Figure S17. ^{13}C-NMR (126 MHz, C_6D_6) of **S5**.

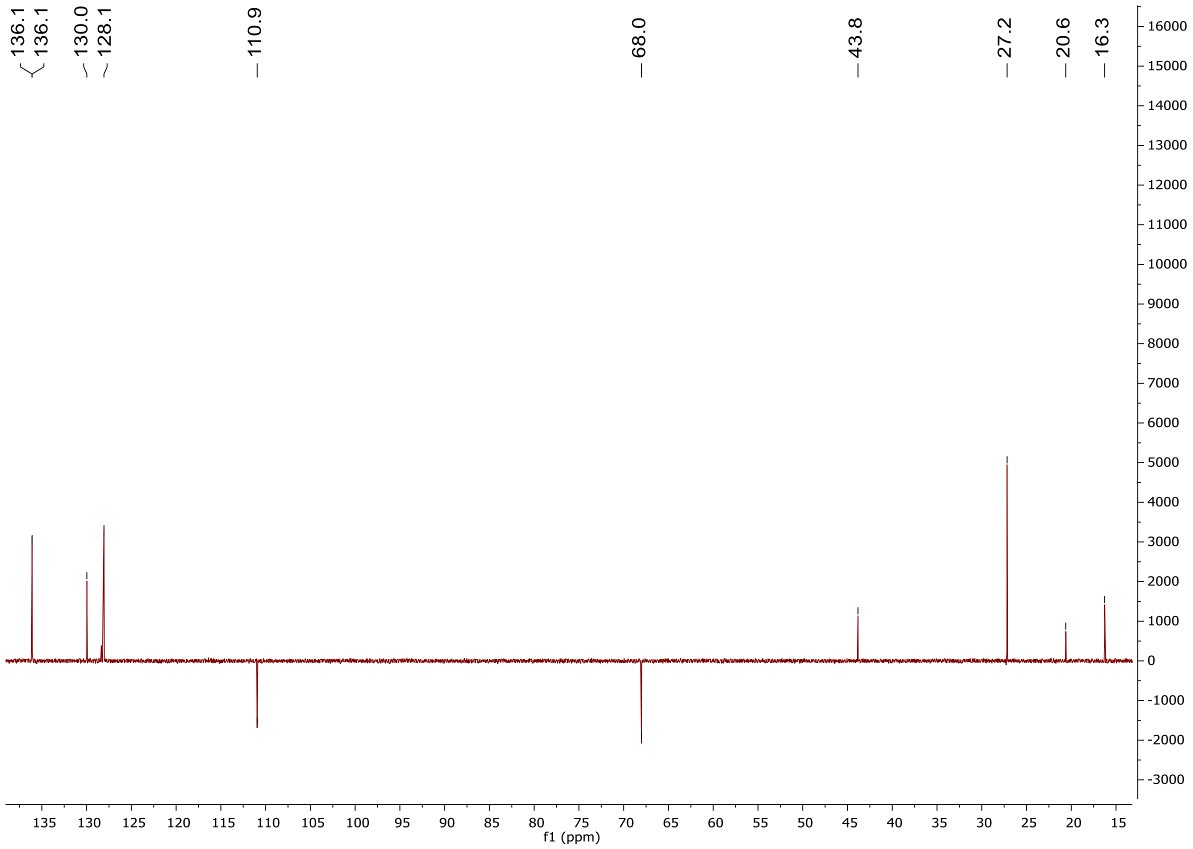

Supplementary Figure S18. DEPT135 (126 MHz, C_6D_6) of **S5**.

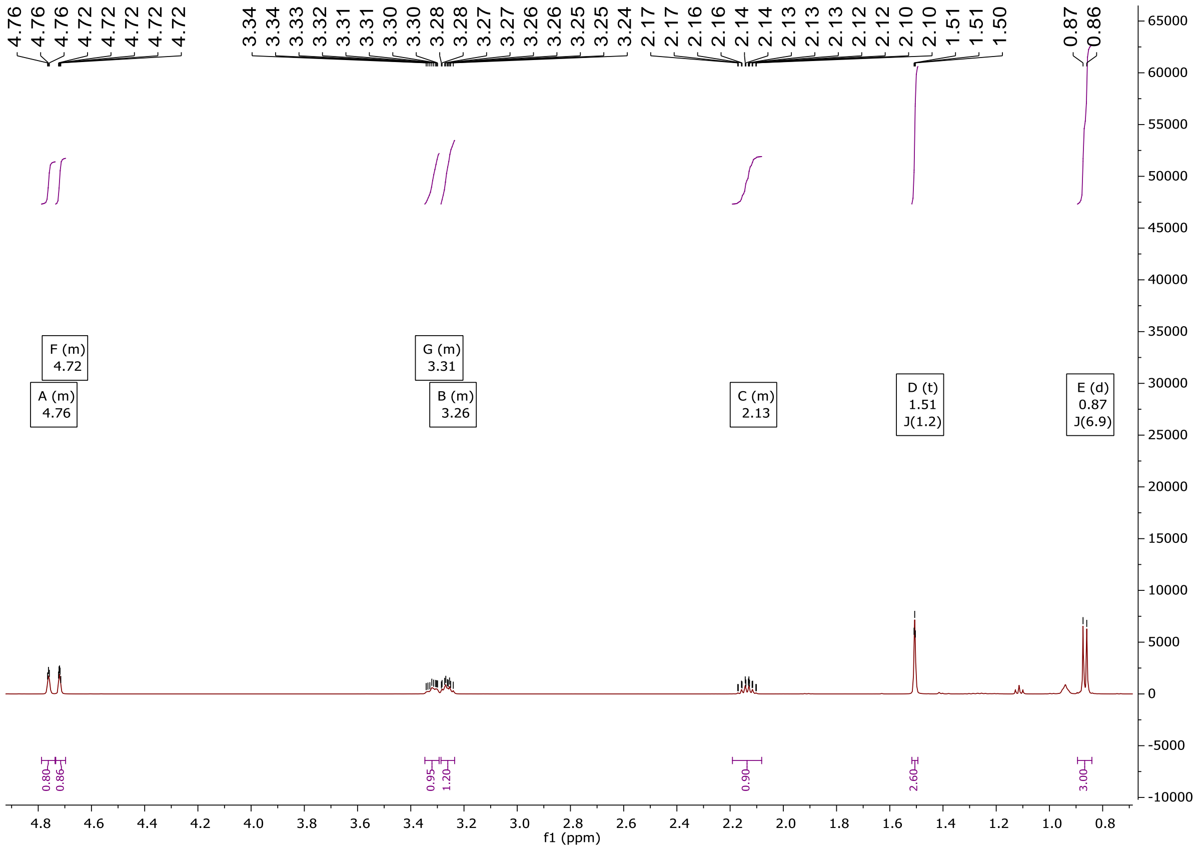

Supplementary Figure S19. ^{1}H-NMR (500 MHz, C_6D_6) of **S6**.

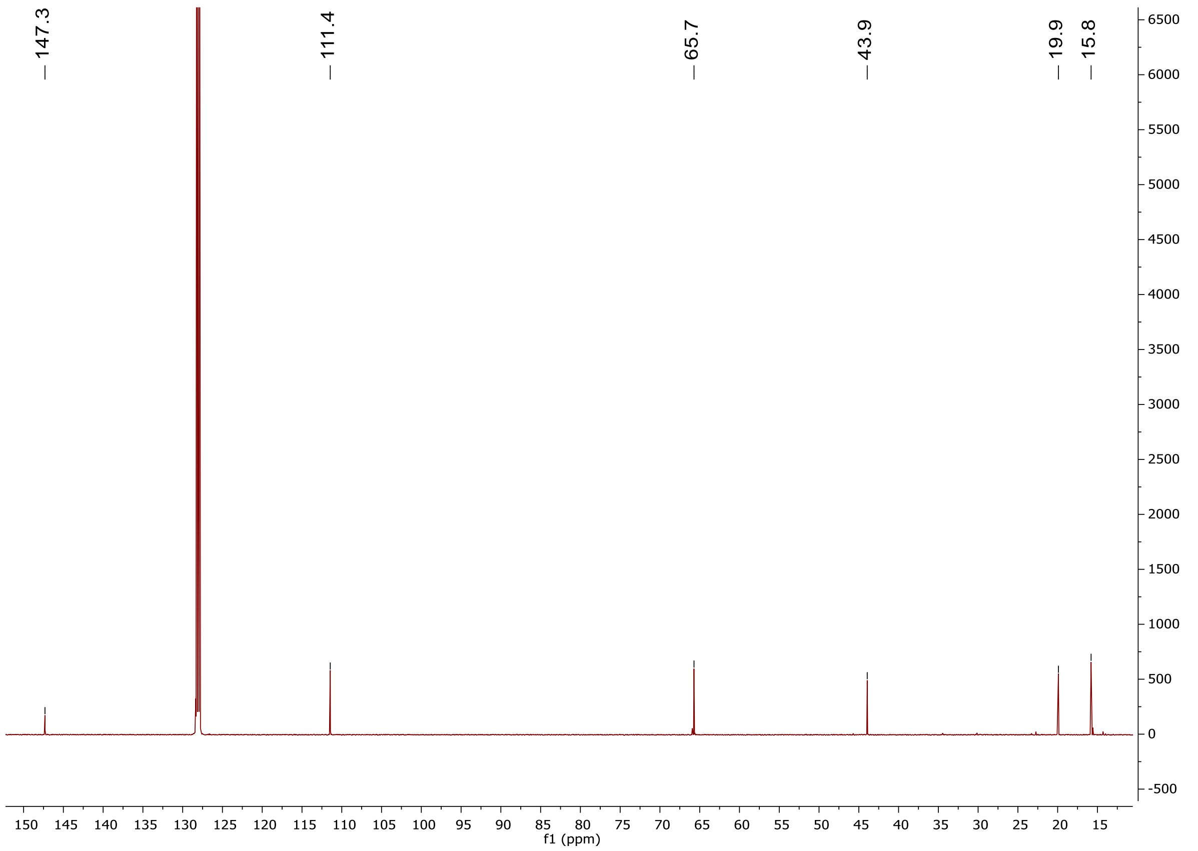

Supplementary Figure S20. ^{13}C-NMR (126 MHz, C_6D_6) of **S6**.

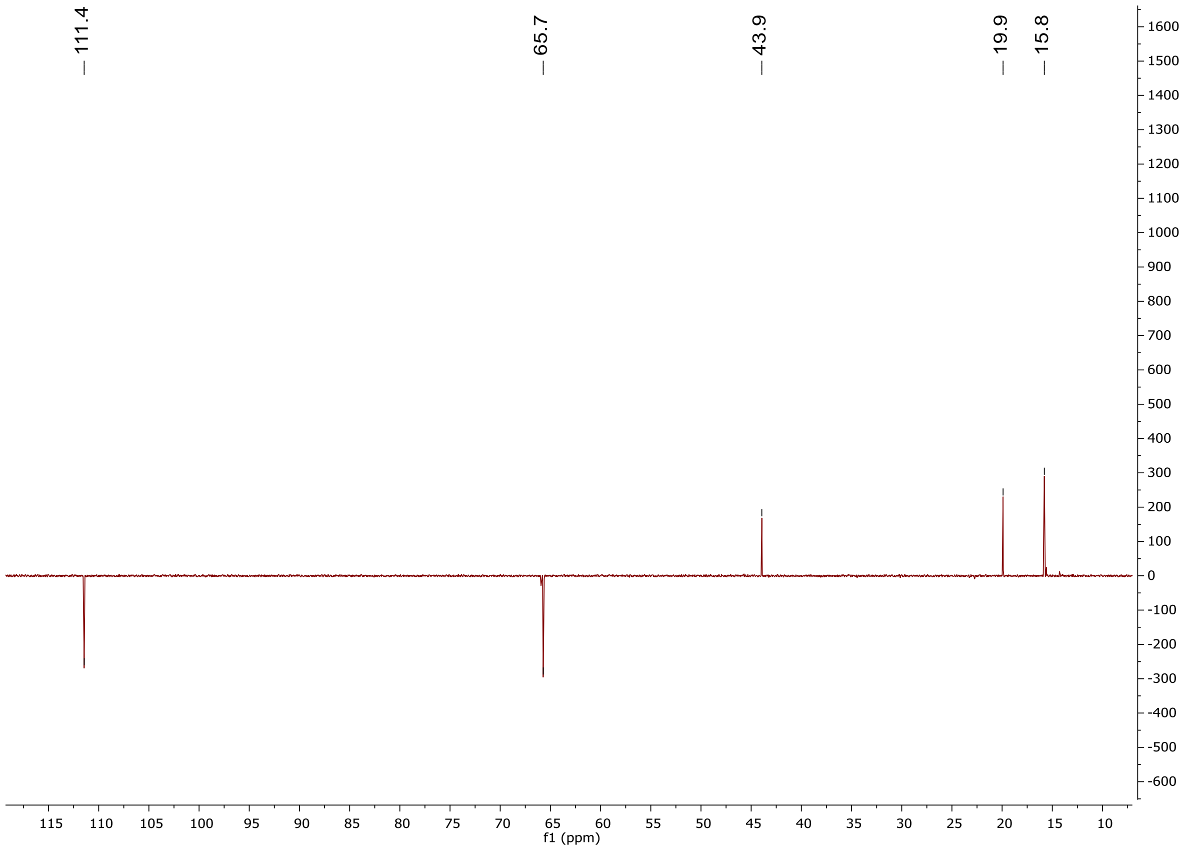

Supplementary Figure S21. DEPT135 (126 MHz, C_6D_6) of **S6**.

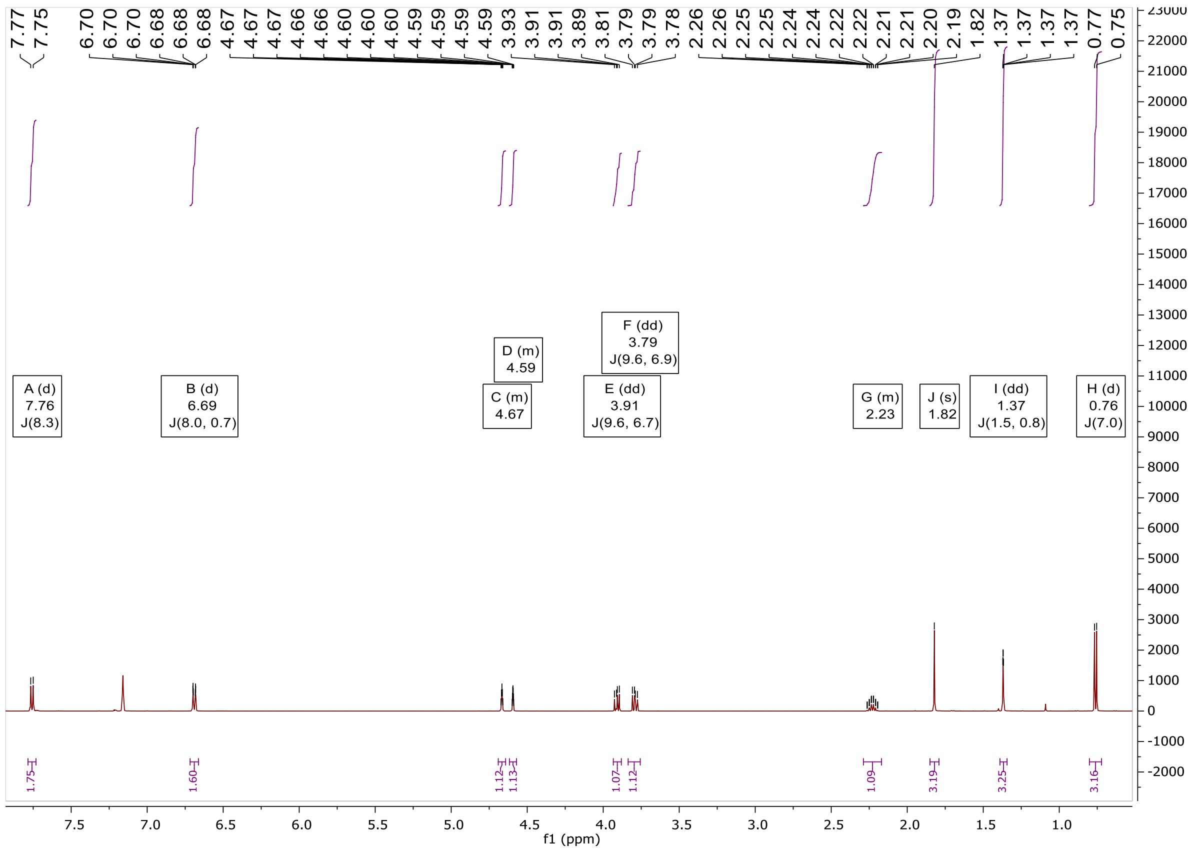

Supplementary Figure S22. ^{1}H-NMR (500 MHz, C_6D_6) of **S7**.

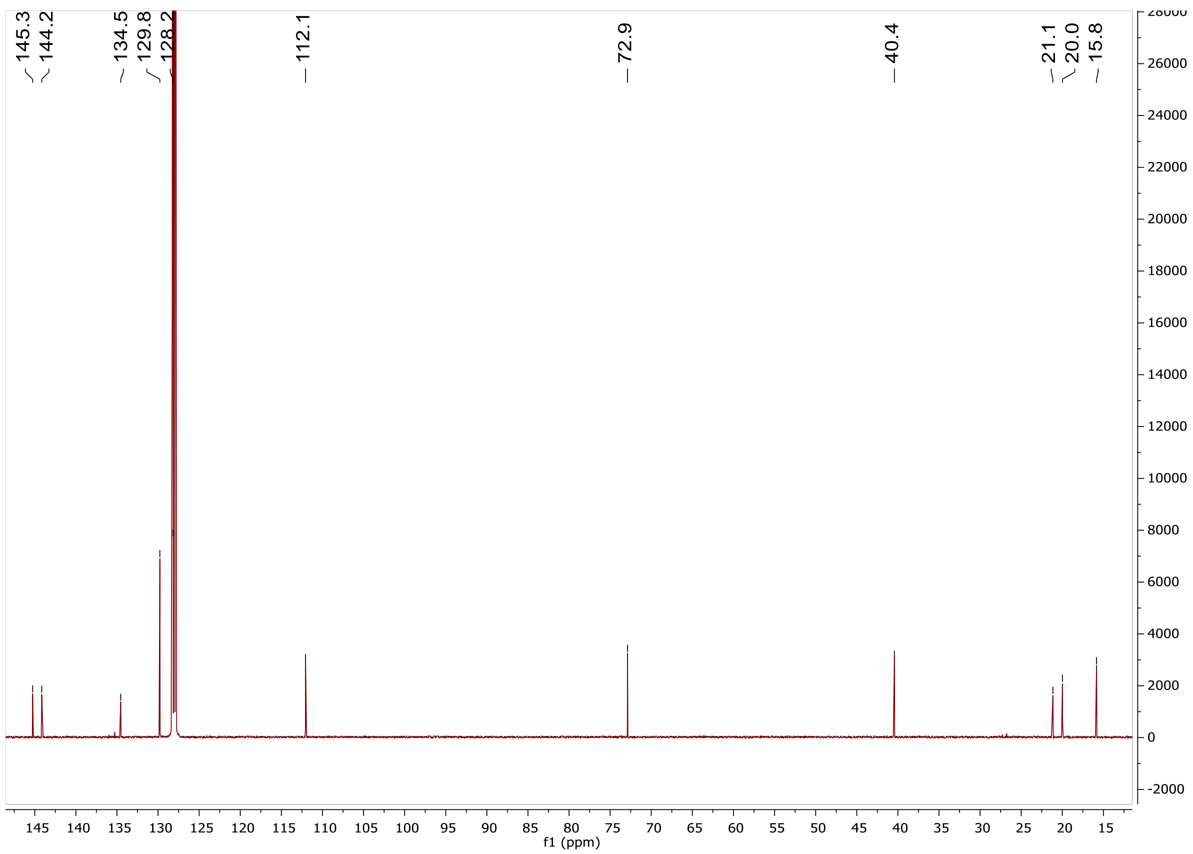

Supplementary Figure S23. ^{13}C-NMR (126 MHz, C_6D_6) of **S7**.

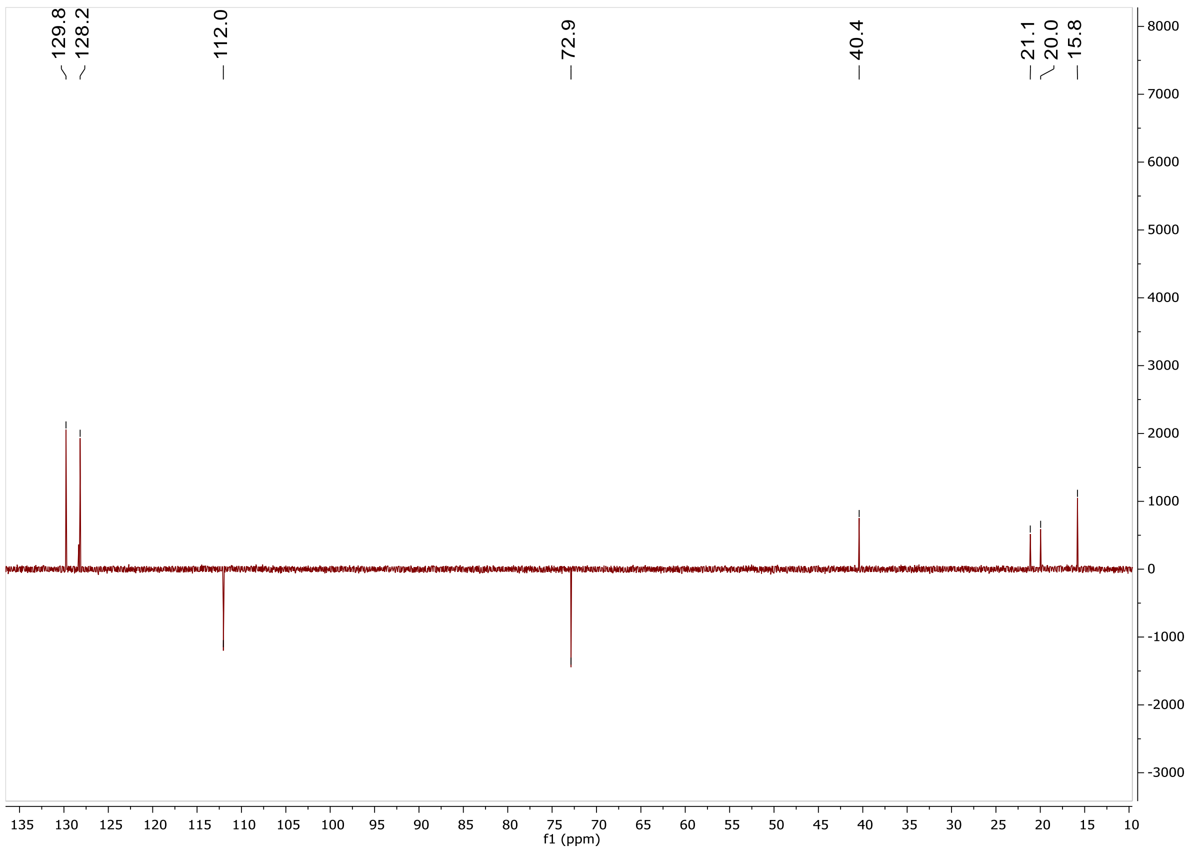

Supplementary Figure S24. DEPT135 (126 MHz, C_6D_6) of **S7**.

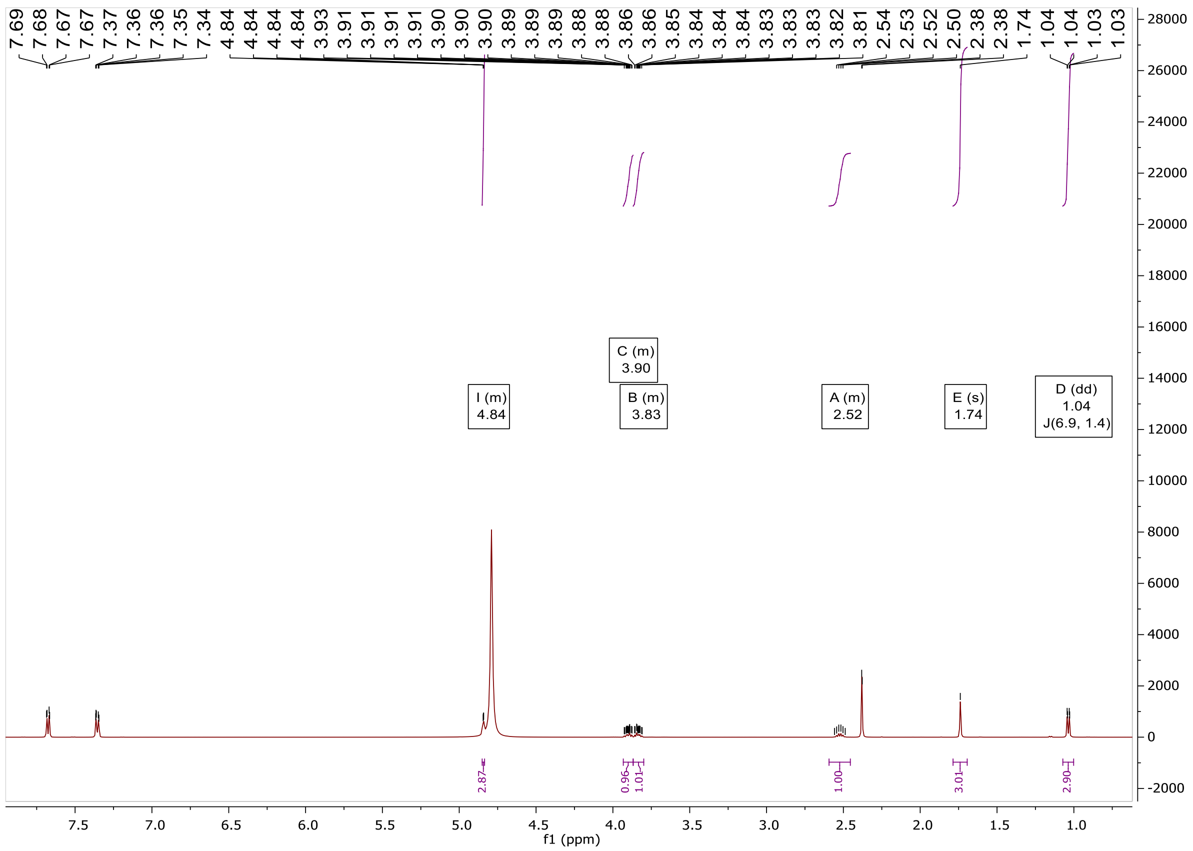

Supplementary Figure S25. ^{1}H-NMR (500 MHz, D_2O) of **S8**.

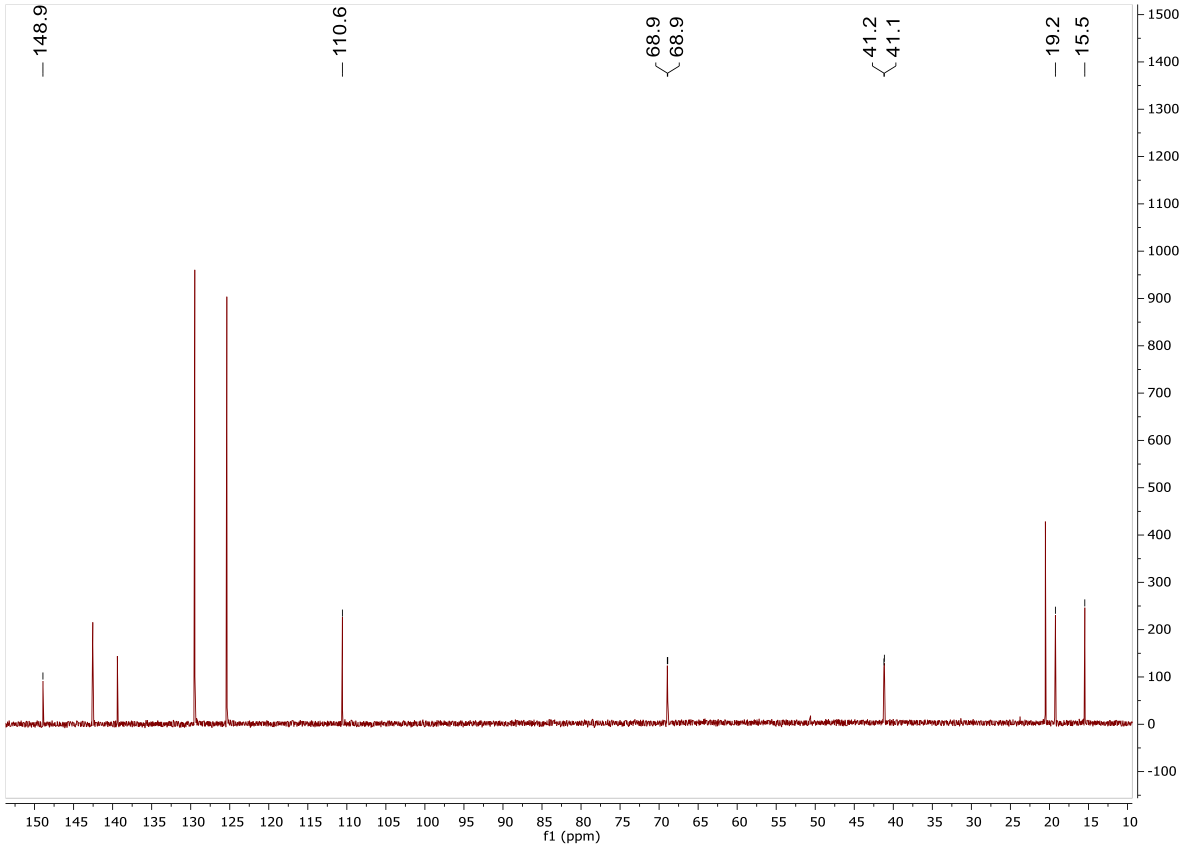

Supplementary Figure S26. ^{13}C-NMR (126 MHz, D_2O) of **S8**.

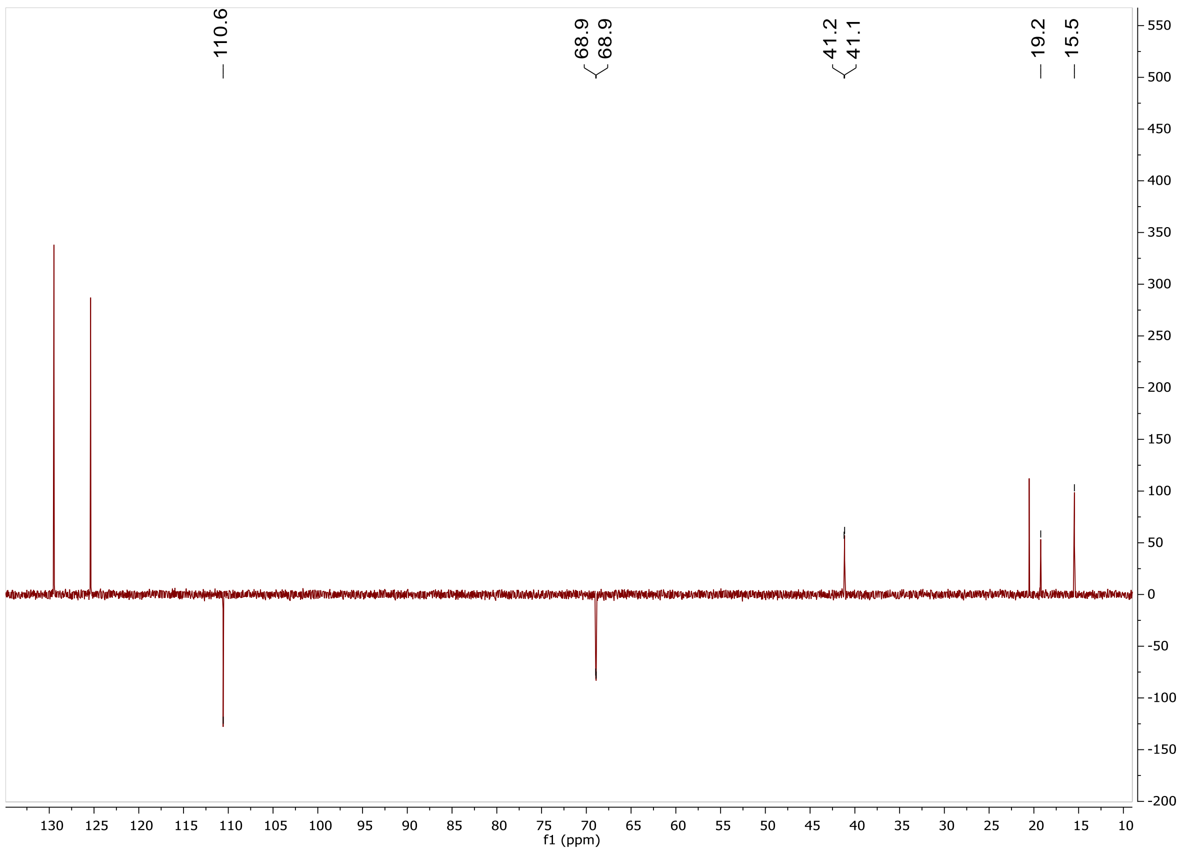

Supplementary Figure S27. DEPT135 (126 MHz, D_2O) of **S8**.

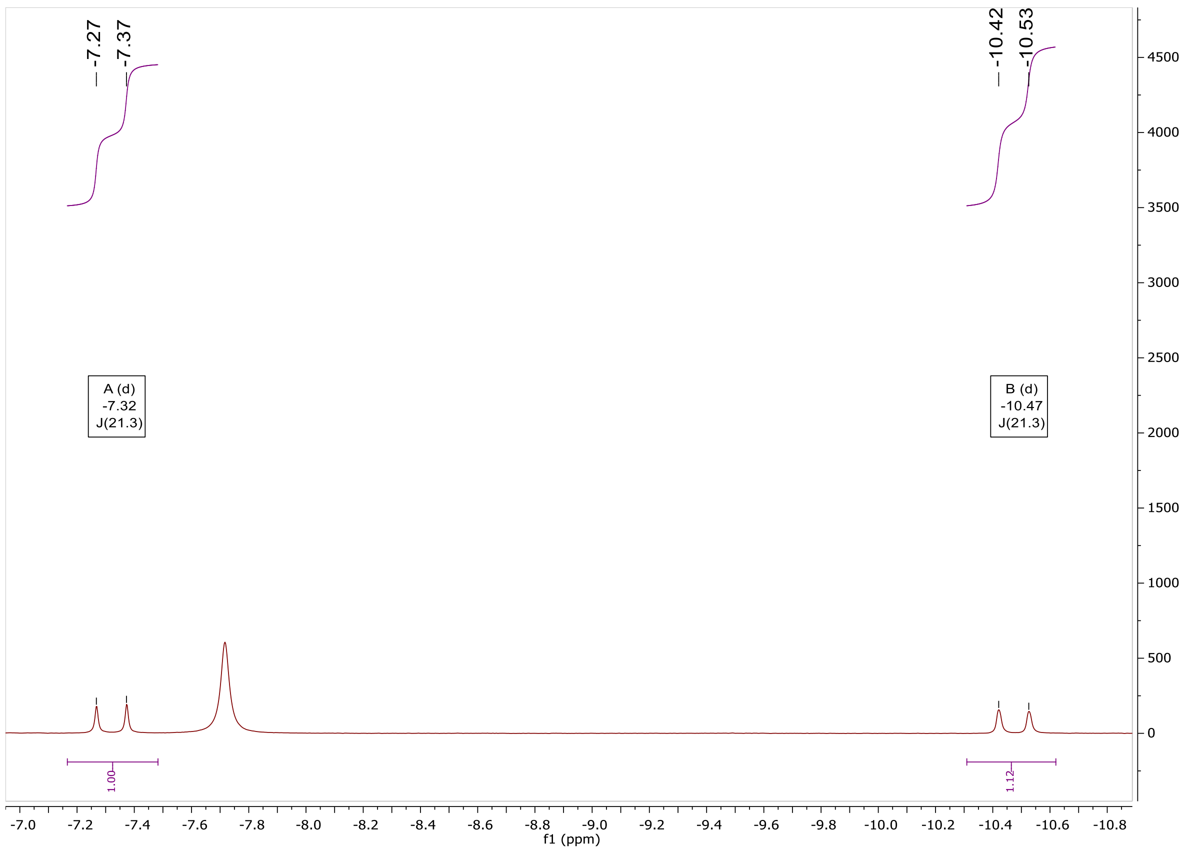

Supplementary Figure S28. ^{13}P-NMR (202 MHz, D_2O) of **S8**.

References

[1] L. Drummond, M. J. Kschowak, J. Breitenbach, H. Wolff, Y. M. Shi, J. Schrader, H. B. Bode, G. Sandmann, M. Buchhaupt, *ACS Synth Biol* **2019**, *8*, 1303-1313.

[2] B. M. Trost, J. P. Papillon, *J Am Chem Soc* **2004**, *126*, 13618-13619.

[3] J. D. White, G. N. Reddy, G. O. Spessard, *J. Am. Chem. Soc.* **1988**, *110*, 1624.

[4] S. Sasaki, S. Samejima, T. Uruga, K. Anzai, N. Nishi, E. Kawakita, K. Takao, K. Tadano, *J Antibiot (Tokyo)* **2013**, *66*, 147-154.

[5] J. D. White, G. N. Reddy, G. O. Spessard, *J Chem Soc, Perkin Trans 1* **1993**, 759-767.

[6] V. J. Davisson, A. B. Woodside, T. R. Neal, K. E. Stremler, M. Muehlbacher, C. D. Poulter, *J Org Chem* **1986**, *51*, 4768-4779.
